# Novel Rivastigmine Derivatives as Promising Multi-Target Compounds for Potential Treatment of Alzheimer’s Disease

**DOI:** 10.3390/biomedicines10071510

**Published:** 2022-06-26

**Authors:** David Vicente-Zurdo, Noelia Rosales-Conrado, M. Eugenia León-González, Leonardo Brunetti, Luca Piemontese, A. Raquel Pereira-Santos, Sandra M. Cardoso, Yolanda Madrid, Sílvia Chaves, M. Amélia Santos

**Affiliations:** 1 Centro de Química Estrutural, Institute of Molecular Sciences, Departamento de Engenharia Química, Instituto Superior Técnico, Universidade de Lisboa, Av. Rovisco Pais 1, 1049-001 Lisboa, Portugal; davidvic@ucm.es (D.V.-Z.); leonardo.brunetti@uniba.it (L.B.); 2 Department of Analytical Chemistry, Faculty of Chemistry, Complutense University of Madrid, Avenida Complutense s/n, 28040 Madrid, Spain; nrosales@ucm.es (N.R.-C.); leongon@ucm.es (M.E.L.-G.); ymadrid@quim.ucm.es (Y.M.); 3 Department of Pharmacy-Pharmaceutical Sciences, University of Bari Aldo Moro, Via E. Orabona 4, 70125 Bari, Italy; luca.piemontese@uniba.it; 4 CNC-Center for Neuroscience and Cell Biology, University of Coimbra, 3000-370 Coimbra, Portugal; arsantos@cnc.uc.pt (A.R.P.-S.); cardoso.sandra.m@gmail.com (S.M.C.); 5 FMUC-Faculty of Medicine, University of Coimbra, 3000-370 Coimbra, Portugal

**Keywords:** Alzheimer’s disease, multi-target drugs, rivastigmine, neurodegenerative, amyloid aggregation, acetylcholinesterase, butyrylcholinesterase

## Abstract

Alzheimer’s disease (AD) is the most serious and prevalent neurodegenerative disorder still without cure. Since its aetiology is diverse, recent research on anti-AD drugs has been focused on multi-target compounds. In this work, seven novel hybrids (RIV–BIM) conjugating the active moiety of the drug rivastigmine (RIV) with 2 isomeric hydroxyphenylbenzimidazole (BIM) units were developed and studied. While RIV assures the inhibition of cholinesterases, BIM provides further appropriate properties, such as inhibition of amyloid β-peptide (Aβ) aggregation, antioxidation and metal chelation. The evaluated biological properties of these hybrids included antioxidant activity; inhibition of acetylcholinesterase (AChE), butyrylcholinesterase (BChE) and Aβ_42_ aggregation; as well as promotion of cell viability and neuroprotection. All the compounds are better inhibitors of AChE than rivastigmine (IC_50_ = 32.1 µM), but compounds of series **5** are better inhibitors of BChE (IC_50_ = 0.9−1.7 µM) than those of series **4**. Series **5** also showed good capacity to inhibit self- (42.1−58.7%) and Cu(II)-induced (40.3−60.8%) Aβ aggregation and also to narrow (22.4−42.6%) amyloid fibrils, the relevant compounds being **5b** and **5d**. Some of these compounds can also prevent the toxicity induced in SH-SY5Y cells by Aβ_42_ and oxidative stress. Therefore, RIV–BIM hybrids seem to be potential drug candidates for AD with multi-target abilities.

## 1. Introduction

Neurodegenerative diseases, such as Alzheimer’s disease (AD), Parkinson’s disease (PD), Huntington’s disease (HD) and amyotrophic lateral sclerosis (ALS), are a group of complex disorders with multiple aetiologies and pathogeneses [1]. The most common cause of dementia is AD, representing 70% of the neurodegenerative diseases and entailing huge financial and healthcare costs [2,3,4]. Nowadays, AD affects around 50 million people worldwide, and this number is expected to increase in the coming years, making AD one of the top six causes of death [5]. AD is an age-related disorder, the main hallmark of which is the formation of senile plaques outside the neurons [6]. Although its origin remains unclear, the most widely accepted explanation is the “amyloid cascade hypothesis” [7,8,9]. In fact, the aggregation of amyloid plaques causes irreversible damage to neurons and synapses, resulting in cognitive deficits. Senile plaques are comprised of fibrillary aggregates of amyloid-beta peptides (Aβ), which enclose elevated levels of Aβ_1-42_ (Aβ_42_), resulting from the proteolytic cleavage of transmembrane proteins called Amyloid Precursor Proteins (APPs) by β- and γ-secretases; Aβ_1-42_ includes 42 amino acid residues from 672 (Asp) to 713 (Ala) of APP [10,11,12]. The process of Aβ_42_ aggregation into amyloid plaques is not yet clearly understood, but several factors, such as metal ion dyshomeostasis and increased oxidative stress, have been reported to trigger their formation [13]. In fact, more than two decades ago several studies revealed the accumulation of metal ions, such as Fe(II), Cu(II) and Zn(II), in amyloid aggregates [14]. Although they have been considered one of the main reasons for the protein aggregation in AD, their role in the pathogenesis remains unresolved, with findings of copper deficiency in some regions of post-mortem brains of AD patients [15]. However, the most critical roles have been attributed to the redox active metals, such as copper and iron, which, through Fenton’s reaction, can produce reactive oxygen species (ROS), induce protein misfold and favour amyloid aggregation [16,17]. Therefore, targeting metal dyshomeostasis is a strategy that has been adopted for potential AD therapy [18,19], and numerous compounds enclosing metal chelating groups have been developed with envisagement of the neutralization of several mechanisms of AD pathogenesis, namely, those associated with oxidative stress in the brain and metal binding to the aberrant amyloid peptide (Aβ) [20,21,22,23,24].

Some neurotransmitters also play an important role in the aetiology of AD. In particular, those of the cholinergic system, acetylcholine (ACh) and butyrylcholine (BCh), are involved in critical neuronal processes, such as memory, learning, sensory information, attention and rest [9]. However, AD also affects the cholinergic system by the interaction between Aβ and the cholinergic receptors, decreasing limbic and neocortical innervation [25,26]. For this reason, inhibitors of cholinesterases (acetylcholinesterase (AChE) and butyrylcholinesterase (BChE)) increase the availability of ACh and BCh (involved in cholinergic signalling), thus improving central nervous system (CNS) performance and leading to symptomatic relief [3,26]. Therefore, up to now, the main class of anti-AD drugs approved by the US Food and Drug Administration (FDA) has been based on inhibitors of cholinesterases. In fact, among the six drugs approved in the US to treat AD, four (tacrine, donepezil, rivastigmine and galantamine) are AChE inhibitors [27,28,29], memantine is a *N*-methyl-D-aspartic acid (NMDA) receptor (NMDAR) antagonist [30] and aduhelm (aducanumab) is a monoclonal antibody (amyloid targeting drug) that has been recently approved (in June 2021) [31].

Although progress has been made in understanding the mechanisms of the disease towards its treatment, all the approved anti-AD drugs have focused on only one disease target and only lead to temporary symptomatic relief. The numerous and complex pathological features of this disease are surely the main reason for the absence of disease-modifying drugs up to now. Therefore, to tackle this multi-factorial disease, the development of multi-target therapeutic agents has been increasingly recognized as a promising strategy to combat AD and many authors have pursued this drug development strategy, in many cases via drug repositioning approaches [22,23,24,32,33,34].

Following our recent multi-target approach for anti-AD drugs, based on the hybridization of tacrine and donepezil [35,36], we set up our research project to develop a novel series of molecular hybrids (RIV–BIMs) by conjugating the rivastigmine pharmacophore (RIV) with a hydroxyphenylbenzimidazole (BIM) moiety. Rivastigmine (trade name Exelon) is a well-known, second-generation carbamate ChE inhibitor drug (approved by the FDA in 2000) that alleviates the symptoms of the disease through the deceleration of its progression [37,38] and presents advantages over first-generation drugs: rapid BBB crossing and low marginal side effects [39]. Although a high number of recently developed multi-target-directed anti-AD drug candidates have included the active moiety of approved AChE inhibitor drugs (e.g., tacrine, donepezil), only a very few have enclosed the pharmacophoric moiety of the drug inhibitor of both ChEs (rivastigmine) [33,40,41]. The selection of hydroxyphenylbenzimidazole (BIM), as the second active moiety of the (RIV–BIM) hybrids, is motivated by its analogy with thioflavin-based intercalation compounds and also by its already demonstrated good capacity to inhibit amyloid aggregation [35,42], reduce ROS and chelate metal ions involved in AD [33,42,43,44]. In fact, recently reported conjugates of BIM derivatives with two anti-AD approved drugs, namely, tacrine and donepezil pharmacophores, were proven to endow the corresponding molecular hybrids with improved AChE inhibitory capacity and other relevant biological activities in the context of well-recognized targets for AD therapy [35,44].

**Figure 1:**
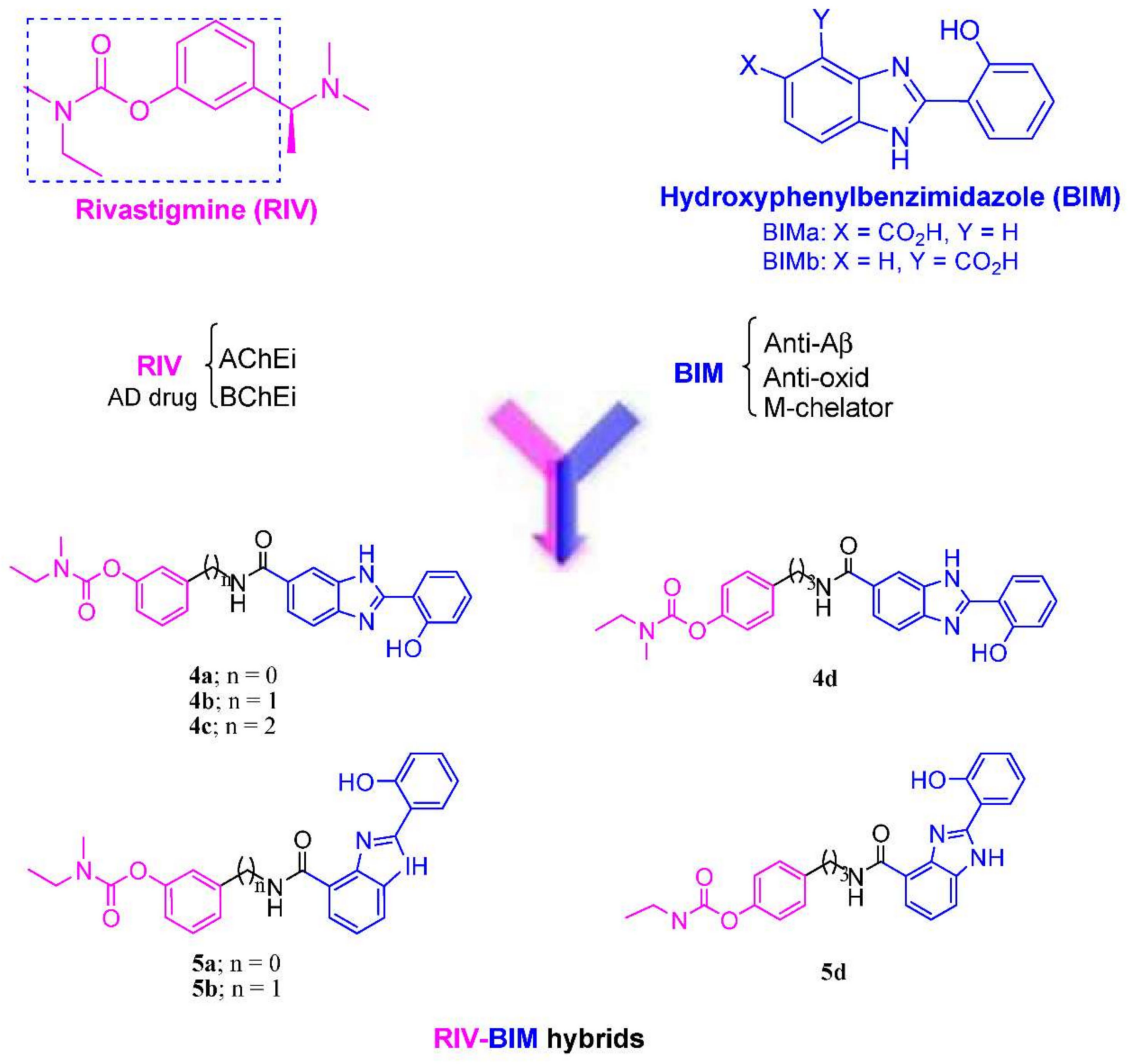
Design strategy for the RIV–BIM hybrids (**4a**–**4d**) and (**5a**–**5d**), from templates with rivastigmine and hydroxyphenylbenzimidazole and positional isomers.

Herein, we report the design, development and study of a new set of seven RIV–BIM conjugates with some isomeric structural variations encompassing different sizes of linkers between the bioactive moieties and the positional isomerization of the BIM moiety. In particular, *para*-substituted (BIMa) and *ortho*-substituted (BIMb) resulted in two respective series (**4** and **5**) of compounds (Figure 1) with some expected differences in their biochemical properties. Molecular modelling for this series of novel potential drug candidates allows predictions of their interactions with enzymes and aids the rationalization of results as well as their pharmacokinetic properties. Specifically, docking studies of the compounds with AChE and BChE, as well as modelling of pharmacokinetic properties, were carried out by using in-house computational tools. Essential biological properties have also been evaluated, such as antioxidant activity (radical scavenging activity), inhibition of AChE and BChE, inhibition of Aβ_42_ self-aggregation and Cu(II)-induced aggregation, cell viability and neuroprotection capacity. The results are discussed in terms of structure–activity relationships, with predictions for the best potential anti-AD drug candidates deserving further study.

## 2. Materials and Methods

### 2.1. Chemistry

#### 2.1.1. General Methods and Materials

Reagents of analytical grade were acquired from Honeywell, Labchem, Sigma-Aldrich and Scharlab. Solvents were dried in consonance with standard methods [45]. TLC was performed to monitor chemical reactions using aluminium plates coated with silica gel 60 F_254_ obtained from Merck. Column chromatographic separations were performed with silica gel 60A 70–200 μ obtained from Carlo Erba Reagents. Melting points (M.P.s) were measured using a Leica Galen III hot stage microscope. NMR spectra (^1^H and ^13^C) were recorded on a Bruker AVANCE III-300 NMR spectrometer (at 300 and 75 MHz). Standard internal reference of tetramethylsilane (TMS) was employed to report the chemical shifts (δ). The abbreviations used are as follows: s = singlet, d = doublet, t = triplet, q = quartet, m = multiplet. Mass spectra (ESI-MS) were captured with a 500 MS LC Ion Trap mass spectrometer (Varian Inc., Palo Alto, CA, USA) equipped with an ESI ion source, operated in the positive ion mode. High-resolution mass spectra (HRMS) were obtained on a Bruker Impact II quadrupole mass spectrometer (Bruker Daltoniks, Billerica, MA, USA).

#### 2.1.2. Preparation of Hydroxyphenylbenzimidazole Carboxylic Acids (BIMa, BIMb)

To a solution of 6 mmol of salicylaldehyde in 10 mL of DMA (*N*,*N*-dimethylacetamide) were added 6 mmol of diaminobenzoic acid (3,4-diaminobenzoic acid or 2,3-diaminobenzoic acid) and 7.2 mmol of Na_2_S_2_O_5_. The mixture was heated to 100 °C for 12 h until the completion of the reaction was confirmed by TLC. The reaction mixture was then cooled at room temperature, diluted with ethyl acetate, dried with Na_2_SO_4_, filtered and concentrated in vacuo. The solid obtained was collected on a sintered-glass filter and washed with cold dichloromethane to provide the desired solid compound.

##### 2-(2-Hydroxyphenyl)-1*H*-benzo[*d*]imidazole-6-carboxylic Acid (BIMa)

This was synthesized from 3,4-diaminobenzoic acid to give **BIMa** as a white-coloured solid. The completion of the reaction was controlled by TLC (CH_2_Cl_2_/MeOH, 15/1). Yield = 79.9%. ^1^H NMR (300 MHz, MeOD-*d*_4_), δ (ppm): 8.29 (s, 1H, BIMa-*Ph*), 7.92–7.97 (m, 2H, BIMa-*Ph*), 7.63 (d, 1H, *J* = 9 Hz, BIMa-*Ph*), 7.35 (t, 1H, *J* = 9 Hz, BIMa-*Ph*), 6.95–7.03 (m, 2H, BIMa-*Ph*). MS-ESI (*m*/*z*): 253.15 (M − 1)^−^, 254.12 (M)^−^, 255.12 (M + 1)^+^, 256.14 (M + 2)^+^.

##### 2-(2-Hydroxyphenyl)-1*H*-benzo[*d*]imidazole-7-carboxylic Acid (BIMb)

This was synthesized from 2,3-diaminobenzoic acid to give **BIMb** as a light-brown-coloured solid. The completion of the reaction was controlled by TLC (CH_2_Cl_2_/MeOH, 2/1). Yield = 86.6%. ^1^H NMR (300 MHz, DMSO-*d*_6_), δ (ppm): 8.34 (d, 1H, *J* = 9 Hz, BIMb-H-6), 7.93 (d, 1H, *J* = 6 Hz, BIMb-*Ph*), 7.84 (d, 1H, *J* = 9 Hz, BIMb-*Ph*), 7.33–7.41 (m, 2H, BIMb-*Ph*), 7.07 (d, 1H, *J* = 9 Hz, BIMb-*Ph*), 7.01 (t, 1H, *J* = 9 Hz, BIMb-*Ph*). MS-ESI (*m*/*z*): 253.17 (M − 1)^−^, 254.14 (M)^−^, 255.04 (M + 1)^+^, 256.08 (M + 2)^+^.

##### 2.1.3. General Procedure for the Synthesis of the Carbamates (**2a**–**d**)

To a solution of 1 mL of TEA (triethylamine) were added 1.65 mmol of *N*-ethylmethylcarbomyl chloride and 1.62 mmol of the nitro- or cyano-derivative (3-nitrophenol, 3-cyanophenol, (3-hydroxyphenyl)acetonitrile or 3-(4-hydroxyphenyl)propionitrile). The mixture was stirred at 95 °C for 12 h. Then, the reaction mixture was diluted with CH_2_Cl_2_, washed with NaOH 1M, dried over Na_2_SO_4_, filtrated and evaporated in vacuo to obtain the desired oiled compound.

##### 3-Nitrophenyl Ethylmethylcarbamate (**2a**)

This was synthesized from 3-nitrophenol, affording **2a** as a pale-brown oil. The completion of the reaction was controlled by TLC (CH_2_Cl_2_/MeOH, 60/1). Yield = 82.9%. ^1^H NMR (300 MHz, MeOD-*d*_4_), δ (ppm): 8.08 (d, 1H, *J* = 9 Hz,-*Ph*), 8.00 (s, 1H, *Ph*), 7.61 (t, 1H, *J* = 9 Hz, *Ph*), 7.53 (d, 1H, *J* = 9 Hz, *Ph*), 3.51 (q, 1H, *J* = 6 Hz, N*CH_2_*CH_3_ rotamer), 3.40 (q, 1H, *J* = 6 Hz, N*CH_2_*CH_3_ rotamer), 3.10 (s, 1.5H, N*CH_3_* rotamer), 2.98 (s, 1.5H, N*CH_3_* rotamer), 1.26 (t, 1.5H, *J* = 6 Hz, CH_2_*CH_3_* rotamer), 1.18 (t, *J* = 6 Hz, CH_2_*CH_3_* rotamer). MS-ESI (*m*/*z*): 225.06 (M + 1)^+^, 225.97 (M + 2)^+^.

##### 3-Cyanophenyl Ethylmethylcarbamate (**2b**)

This was synthesized from 3-cyanophenol, affording **2b** as a pale-brown oil. The completion of the reaction was controlled by TLC (CH_2_Cl_2_/MeOH, 60/1). Yield = 93.1%. ^1^H NMR (300 MHz, MeOD-*d_4_*), δ (ppm): 7.56 (s, 1H, *Ph*), 7.51–7.53 (m, 2H, *Ph*), 7.42 (d, 1H, *J* = 6 Hz, *Ph*), 3.49 (q, 1H, *J* = 9 Hz, N*CH_2_*CH_3_ rotamer), 3.37 (*q*, 1H, *J* = 9 Hz, N*CH_2_*CH_3_ rotamer), 3.08 (s, 1.5H, N*CH_3_* rotamer), 2.96 (s, 1.5H, N*CH_3_* rotamer), 1.23 (*t*, 1.5H, *J* = 9 Hz, CH_2_*CH_3_* rotamer), 1.16 (t, 1.5H, *J* = 9 Hz, CH_2_*CH_3_* rotamer). MS-ESI (*m*/*z*): 205.13 (M + 1)^+^.

##### 3-(Cyanomethyl)phenyl Ethylmethylcarbamate (**2c**)

This was synthesized from (3-hydroxyphenyl)acetonitrile, affording **2c** as a pale-brown oil. The completion of the reaction was controlled by TLC (CH_2_Cl_2_/MeOH, 30/1). Yield = 76.7%. ^1^H NMR (300 MHz, MeOD-*d*_4_), δ (ppm): 7.38 (t, 1H, *J* = 9 Hz, *Ph*), 7.21 (d, 1H, *J* = 9 Hz, *Ph*), 7.10 (s, 1H, *Ph*), 7.06 (d, 1H, *J* = 9 Hz, *Ph*), 3.90 (s, 2H, Ph*CH_2_*CN), 3.49 (q, 1H, *J* = 6 Hz, N*CH_2_*CH_3_ rotamer), 3.37 (q, 1H, *J* = 6 Hz, N*CH_2_*CH_3_ rotamer), 3.08 (s, 1.5H, N*CH_3_* rotamer), 2.96 (s, 1.5H, N*CH_3_* rotamer), 1.24 (*t*, 1.5H, *J* = 6 Hz, CH_2_*CH_3_* rotamer), 1.16 (t, 1.5H, *J* = 6 Hz, CH_2_*CH_3_* rotamer). MS-ESI (*m*/*z*): 219.16 (M + 1)^+^, 220.15 (M + 2)^+^.

##### 4-(2-Cyanoethyl)phenyl Ethylmethylcarbamate (**2d**)

This was synthesized from 3-(4-hydroxyphenyl)propionitrile, affording **2d** as a pale-brown oil. The completion of the reaction was controlled by TLC (CH_2_Cl_2_/MeOH, 30/1). Yield = 99.4%. ^1^H NMR (300 MHz, MeOD-*d*_4_), δ (ppm): 7.30 (d, 2H, *J* = 6 Hz, *Ph*), 7.05 (d, 2H, *J* = 6 Hz, *Ph*), 3.50 (q, 1H, *J* = 6 Hz, N*CH_2_*CH_3_ rotamer), 3.38 (q, 1H, *J* = 6 Hz, N*CH_2_*CH_3_ rotamer), 3.08 (s, 1.5H, N*CH_3_* rotamer), 2.96 (s, 1.5H, N*CH_3_* rotamer), 2.92 (d, 2H, *J* = 6 Hz, Ph*CH_2_*CH_2_), 2.72 (t, 2H, *J* = 6 Hz, CH_2_*CH_2_*CN), 1.25 (t, 1.5H, *J* = 6 Hz, CH_2_*CH_3_* rotamer), 1.17 (t, 1.5H, *J* = 6 Hz, CH_2_*CH_3_* rotamer). MS-ESI (*m*/*z*): 233.17 (M + 1)^+^, 234.22 (M + 2)^+^.

#### 2.1.4. General Procedure for the Synthesis of the Amino-Carbamates (**3a**–**d**)

In this procedure, 1.45 mmol of the nitro- or cyano-carbamate derivatives (**2a–d**) (3-nitrophenyl ethylmethylcarbamate, 3-cyanophenyl ethylmethylcarbamate, 3-(cyanomethyl)phenyl ethylmethylcarbamate, 4-(2-cyanoethyl)phenylethylmethylcarbamate) was hydrogenated in 15 mL of MeOH in the presence of 2.12 mmol of 10% Pd-C for 4 h at 4 bar. Then, the catalyst was filtered off and the solution was evaporated until dryness, providing the final amine as an oil of compounds **3a** and **3b**. For the hydrogenolysis of compounds **3c** and **3d**, an identical procedure was followed, though some drops of concentrated HCl were added to the methanolic suspension. Then, the catalyst was filtered off and the solution was evaporated to dryness. The residue was diluted with CH_2_Cl_2_ and washed with NaOH 1 M and H_2_O until a pH > 7 was reached. The organic layer phase was dried over Na_2_SO_4_ and evaporated. The crude oil compound was purified by chromatography column (with eluent MeOH/CH_2_Cl_2_/NH_3_, 49/49/2).

##### 3-Aminophenyl Ethylmethylcarbamate (**3a**)

This was synthesized from 3-nitrophenyl ethylmethylcarbamate (**2a**) to give **3a** as a pale-yellow oil. The completion of the reaction was controlled by TLC (CH_2_Cl_2_/MeOH, 60/1). Yield = 40.8%. ^1^H NMR (300 MHz, MeOD-*d*_4_), δ (ppm): 7.04 (t, 1H, *J* = 9 Hz, *Ph*), 6.53 (d, 1H, *J* = 9 Hz, *Ph*), 6.40 (s, 1H, *Ph*), 6.35 (d, 1H, *J* = 9 Hz, *Ph*), 3.42 (q, 1H, *J* = 6 Hz, N*CH_2_*CH_3_ rotamer), 3.35 (q, 1H, *J* = 6 Hz, N*CH_2_*CH_3_ rotamer), 3.03 (s, 1.5H, N*CH_3_* rotamer), 2.93 (s, 1.5H, N*CH_3_* rotamer), 1.21 (t, 1.5H, *J* = 6 Hz, CH_2_*CH_3_* rotamer), 1.14 (t, 1.5H, *J* = 6 Hz, CH_2_*CH_3_* rotamer). MS-ESI (*m*/*z*): 195.05 (M + 1)^+^, 196.09 (M + 2)^+^.

##### 3-(Aminomethyl)phenyl Ethylmethylcarbamate (**3b**)

This was synthesized from 3-cyanophenyl ethylmethylcarbamate (**2b**) to give the crude oil of the compound. The completion of the reaction was controlled by TLC (EtOAc/NH3, 98/2). Then, the crude oil compound was purified by chromatography column (with eluent EtOAc/NH3, 98/2), affording the pure compound **3b** as a pale-yellow oil. Yield = 76.6%. ^1^H NMR (300 MHz, MeOD-*d*_4_), δ (ppm): 7.35 (t, 1H, *J* = 9 Hz, *Ph*), 7.21 (d, 1H, *J* = 9 Hz, *Ph*), 7.10 (s, 1H, *Ph*), 7.01 (d, 1H, *J* = 9Hz, *Ph*), 3.85 (s, 2H, Ph*CH_2_*NH_2_), 3.49 (q, 1H, *J* = 6 Hz, N*CH_2_*CH_3_ rotamer), 3.37 (q, 1H, *J* = 6 Hz, N*CH_2_*CH_3_ rotamer), 3.08 (s, 1.5H, N*CH_3_* rotamer), 2.96 (s, 1.5H, N*CH_3_* rotamer), 1.24 (t, 1.5H, *J* = 6 Hz, CH_2_*CH_3_* rotamer), 1.16 (t, 1.5H, *J* = 6 Hz, CH_2_*CH_3_* rotamer). MS-ESI (*m*/*z*): 209.00 (M + 1)^+^, 209.83 (M + 2)^+^.

##### 3-(2-Aminoethyl)phenyl Ethylmethylcarbamate (**3c**)

This was synthesized from 3-(cyanomethyl)phenyl ethylmethylcarbamate (**2c**), affording the pure compound **3c** as a beige oil. Yield = 22.0%. ^1^H NMR (300 MHz, MeOD-*d*_4_), δ (ppm): 7.30 (t, 1H, *J* = 6 Hz, *Ph*), 7.08 (d, 1H, *J* = 6 Hz, *Ph*), 6.95 (s, 1H, *Ph*), 6.94 (d, 1H, *J* = 6 Hz, *Ph*), 3.49 (q, 1H, *J* = 6 Hz, N*CH_2_*CH_3_ rotamer), 3.38 (q, 1H, *J* = 6 Hz, N*CH_2_*CH_3_ rotamer), 3.08 (s, 1.5H, N*CH_3_* rotamer), 2.96 (s, 1.5H, N*CH_3_* rotamer), 2.88 (t, 2H, *J* = 6 Hz, CH_2_*CH_2_*NH_2_), 2.76 (t, 2H, *J* = 6 Hz, Phe*CH_2_*CH_2_), 1.25 (t, 1.5H, *J* = 6 Hz, CH_2_*CH_3_* rotamer), 1.17 (t, 1.5H, *J* = 6 Hz, CH_2_*CH_3_* rotamer). MS-ESI (*m*/*z*): 222.49 (M)^-^, 223.20 (M + 1)^+^, 224.24 (M + 2)^+^.

##### 4-(3-Aminopropyl)phenyl Ethylmethylcarbamate (**3d**)

This was synthesized from 4-(2-cyanoethyl)phenyl ethylmethylcarbamate (**2d**), affording the pure compound **3d** as a pale-yellow oil. Yield = 30.2%. ^1^H NMR (300 MHz, MeOD-*d*_4_), δ (ppm): 7.23 (d, 2H, *J* = 6 Hz, *Ph*), 7.01 (d, 2H, *J* = 6 Hz, *Ph*), 3.49 (q, 1H, *J* = 6 Hz, N*CH_2_*CH_3_ rotamer), 3.38 (q, 1H, *J* = 6 Hz, N*CH_2_*CH_3_ rotamer), 3.07 (s, 1.5H, N*CH_3_* rotamer), 2.89–2.96 (m, 3.5H, Ph*CH_2_*CH_2_ and N*CH_3_* rotamer), 2.71 (t, 2H, *J* = 6 Hz, CH_2_*CH_2_*NH_2_), 1.96 (quintet, 2H, *J* = 6 Hz, CH_2_*CH_2_*CH_2_), 1.24 (t, 1.5H, *J* = 6 Hz, CH_2_*CH_3_* rotamer), 1.16 (t, 1.5H, *J* = 6 Hz, CH_2_*CH_3_* rotamer). MS-ESI (*m*/*z*): 237.20 (M + 1)^+^, 238.18 (M + 2)^+^.

#### 2.1.5. General Synthetic Procedure for the RIV–BIMa Hybrids (**4a**–**d**)

To a water-ice-cooled solution of 1 mmol of 2-(2-hydroxyphenyl)-1*H*-benzo[*d*]imidazole-6-carboxylic acid (**BIMa**) in dry DMF (5 mL), under nitrogen atmosphere, were added 2 mmol of NMM, followed by the addition of 1 mmol of TBTU, and this solution was stirred for 50 min. Then, the carboxylic acid derivative solution was added dropwise to a water-ice-cooled solution of 1 mmol of the corresponding amine derivative (3-aminophenyl ethylmethylcarbamate (**3a**), 3-(aminomethyl)phenyl ethylmethylcarbamate (**3b**), 3-(2-aminoethyl)phenyl ethylmethylcarbamate (**3c**), 4-(3-aminopropyl)phenyl ethylmethylcarbamate (**3d**)) in 5 mL of dry DMF. The reaction mixture was stirred for 20 h under nitrogen atmosphere to reaction completion. Afterwards, DMF was evaporated under high vacuum. The residue was diluted with CH_2_Cl_2_, and the organic solution was washed with H_2_O, dried with Na_2_SO_4_, filtrated, roto-evaporated and dried in vacuo to obtain the crude solid.

##### 3-(2-(2-Hydroxyphenyl)-1*H*-benzo[*d*]imidazole-6-carboxamido)phenyl Ethylmethylcarbamate, RIV–BIMa (**4a**)

This was synthesized from 3-aminophenylethylmethylcarbamate (**3a**). The completion of the reaction was controlled by TLC (AcOEt/Hexane, 5/1). The crude solid was purified by chromatography column (with eluent AcOEt/hexane, 5/1), affording the pure compound **4a** as a light-pink-coloured solid. Yield = 21.4%. M.P. = 200 °C. RF (AcOEt/hexane, 5/1) = 0.8. ^1^H NMR (300 MHz, MeOD-*d*_4_), δ (ppm): 8.22 (s, 1H, *Ph*), 7.95 (d, 1H, *J* = 9 Hz, *Ph*), 7.88 (d, 1H, *J* = 9 Hz, *Ph*), 7.70 (d, 1H, *J* = 9 Hz, *Ph*), 7.63 (s, 1H, *Ph*), 7.55 (d, 1H, *J* = 9 Hz, *Ph*), 7.32–7.40 (m, 2H, *Ph*), 6.97–7.04 (m, 2H, *Ph*), 6.88 (d, 1H, *J* = 9 Hz, *Ph*), 3.51 (q, 1H, *J* = 6 Hz, N*CH_2_*CH_3_ rotamer), 3.40 (q, 1H, *J* = 6 Hz, N*CH_2_*CH_3_ rotamer), 3.10 (s, 1.5H, N*CH_3_* rotamer), 2.98 (s, 1.5H, N*CH_3_* rotamer), 1.26 (t, 1.5H, *J* = 6 Hz, CH_2_*CH_3_* rotamer), 1.18 (t, 1.5H, *J* = 6 Hz, CH_2_*CH_3_* rotamer). ^13^C NMR (75 MHz, MeOD-*d*_4_), δ (ppm): 169.18, 159.69, 153.16, 141.31, 133.55, 131.02, 130.46, 127.80, 123.95, 120.70, 118.94, 118.47, 115.68, 113.94, 45.30, 34.41, 13.55. HRMS (ESI) calculated for C_24_H_23_N_4_O4 [M + H] 431.1719, found 431.1732.

##### 3-((2-(2-Hydroxyphenyl)-1*H*-benzo[*d*]imidazole-6-carboxamido)methyl) Phenyl Ethylmethylcarbamate, RIV–BIMa (**4b**)

This was synthesized from 3-(aminomethyl)phenyl ethylmethylcarbamate (**3b**). The completion of the reaction was controlled by TLC (AcOEt/hexane, 5/1). The crude solid was purified by chromatography column (with eluent AcOEt/hexane, 5/1), affording the pure compound **4b** as a white-coloured solid. Yield = 56.8%. M.P. = 230 °C. RF (AcOEt/hexane, 5/1) = 0.66. ^1^H NMR (300 MHz, MeOD-*d*_4_), δ (ppm): 8.15 (s, 1H, *Ph*), 7.94 (d, 1H, *J* = 9 Hz, *Ph*), 7.80 (d, 1H, *J* = 9 Hz, *Ph*), 7.66 (d, 1H, *J* = 9 Hz, *Ph*), 7.32–7.38 (m, 2H, *Ph*), 7.25 (d, 1H, *J* = 6 Hz, *Ph*), 7.12 (s, 1H, *Ph*), 6.96–7.03 (m, 3H, *Ph*), 4.61 (s, 2H, Ph*CH_2_*NH), 3.48 (q, 1H, *J* = 6 Hz, N*CH_2_*CH_3_ rotamer), 3.37 (q, 1H, *J* = 6 Hz, N*CH_2_*CH_3_ rotamer), 3.07 (s, 1.5H, N*CH_3_* rotamer), 2.95 (s, 1.5H, N*CH_3_* rotamer), 1.23 (t, 1.5H, *J* = 6 Hz, CH_2_*CH_3_* rotamer), 1.15 (t, 1.5H, *J* = 6 Hz, CH_2_*CH_3_* rotamer). ^13^C NMR (75 MHz, MeOD-*d*_4_), δ (ppm): 159.61, 133.20, 130.48, 127.53, 125.70, 120.52, 118.37, 114.11, 44.30, 42.01, 17.38. HRMS (ESI) calculated for C_25_H_25_N_4_O_4_ [M + H] 445.1876, found 445.1897.

##### 3-(2-(2-(2-Hydroxyphenyl)-1*H*-benzo[*d*]imidazole-6-carboxamido)ethyl) Phenylethylmethylcarbamate, RIV–BIMa (**4c**)

This was synthesized from 3-(2-aminoethyl)phenyl ethylmethylcarbamate (**3c**). The completion of the reaction was controlled by TLC (AcOEt/hexane, 5/1). The crude solid was purified by chromatography column (with eluent AcOEt/hexane, 5/1), affording the pure compound **4c** as a white-coloured solid. Yield = 73.9%. M.P. = 212 °C. RF (AcOEt/hexane, 5/1) = 0.64. ^1^H NMR (300 MHz, CD_3_OD-*d*_4_), δ (ppm): 8.05 (s, 1H, *Ph*), 7.94 (d, 1H, *J* = 9 Hz, *Ph*), 7.72 (d, 1H, *J* = 9 Hz, *Ph*), 7.63 (d, 1H, *J* = 9 Hz, *Ph*), 7.28–7.38 (m, 2H, *Ph*), 7.14 (d, 1H, *J* = 9 Hz, *Ph*), 6.94–7.03 (m, 4H, *Ph*), 3.64 (t, 2H, *J* = 9 Hz, CH_2_*CH_2_*NH), 3.47 (q, 1H, *J* = 6 Hz, N*CH_2_*CH_3_ rotamer), 3.37 (q, 1H, *J* = 6 Hz, N*CH_2_*CH_3_ rotamer), 3.06 (s, 1.5H, N*CH_3_* rotamer), 2.93–2.98 (m, 3.5H, Ph*CH_2_*CH_2_ and N*CH_3_* rotamer), 1.20 (t, 1.5H, *J* = 6 Hz, CH_2_*CH_3_* rotamer), 1.14 (t, 1.5H, *J* = 6 Hz, CH_2_*CH_3_* rotamer). ^13^C NMR (75 MHz, MeOD-*d*_4_), δ (ppm): 170.69, 159.61, 152.93, 142.51, 133.17, 130.43, 127.50, 127.22, 123.44, 120.90, 120.51, 118.36, 114.12, 45.15, 42.53, 36.26. HRMS (ESI) calculated for C_26_H_27_N_4_O_4_ [M + H] 459.2032, found 459.2049.

##### 4-(3-(2-(2-Hydroxyphenyl)-1*H*-benzo[*d*]imidazole-6-carboxamido)propyl) Phenyl Ethylmethylcarbamate, RIV–BIMa (**4d**)

This was synthesized from 4-(3-aminopropyl)phenylethylmethylcarbamate (**3d**). The completion of the reaction was controlled by TLC (CH_2_Cl_2_/MeOH, 15/1). The crude solid was purified by chromatography column (with eluent CH_2_Cl_2_/MeOH, 15/1), affording the pure compound **4d** as a light-yellow-coloured solid. Yield = 29.3%. M.P. = 231 °C. RF (AcOEt/hexane, 5/1) = 0.64. ^1^H NMR (300 MHz, MeOD-*d*_4_), δ (ppm): 8.08 (s, 1H, *Ph*), 7.94 (d, 1H, *J* = 6 Hz, *Ph*), 7.74 (d, 1H, *J* = 6 Hz, *Ph*), 7.64 (d, 1H, *J* = 9 Hz, *Ph*), 7.36 (t, 1H, *J* = 9 Hz, *Ph*), 7.25 (d, 2H, *J* = 9 Hz, *Ph*), 6.97–7.03 (m, 4H, *Ph*), 3.42–3.47 (m, 3H, CH_2_*CH_2_*NH and N*CH_2_*CH_3_ rotamer), 3.34 (q, 1H, *J* = 6 Hz, N*CH_2_*CH_3_ rotamer), 3.06 (s, 1.5H, N*CH_3_* rotamer), 2.95 (s, 1.5H, N*CH_3_* rotamer), 2.72 (t, 2H, *J* = 9 Hz, Ph*CH_2_*CH_2_), 1.96 (quintet, 2H, *J* = 9 Hz, CH_2_*CH_2_*CH_2_), 1.22 (t, 1.5H, *J* = 9 Hz, CH_2_*CH_3_* rotamer), 1.18 (t, 1.5H, *J* = 9 Hz, CH_2_*CH_3_* rotamer). ^13^C NMR (75 MHz, DMSO-*d*_6_), δ (ppm): 173.90, 166.43, 158.04, 153.75, 149.38, 138.60, 132.18, 129.04, 126.57, 121.71, 119.31, 117.30, 112.49, 48.64, 43.47, 33.91, 33.59, 32.03, 31.06, 29.34, 13.10, 12.31. HRMS (ESI) calculated for C_25_H_25_N_4_O_4_ [M + H] 473.2189, found 473.2203.

#### 2.1.6. General Procedure for the RIV–BIMb Hybrids (**5a**, **5b**, **5d**)

To a water-ice-cooled solution of 1 mmol of 2-(2-hydroxyphenyl)-1*H*-benzo[*d*]imidazole-7-carboxylic acid (BIMb), under nitrogen atmosphere, was added 1 mmol of HOBT, followed by the addition of 1 mmol of EDCI, which was then stirred for 50 min. This acid solution was added dropwise to a water-ice-cooled solution of 1 mmol of the corresponding amine (3-aminophenyl ethylmethylcarbamate (3a), 3-(aminomethyl)phenyl ethylmethylcarbamate (3b), 4-(3-aminopropyl)phenyl ethylmethylcarbamate (3d)) in 5 mL of dry DMF. The reaction mixture was stirred for 20 h under nitrogen atmosphere. DMF was then evaporated under high vacuum. The residue was diluted with CH_2_Cl_2_, washed with H_2_O, dried with Na_2_SO_4_, filtrated and dried to obtain the crude solid.

##### 3-(2-(2-Hydroxyphenyl)-1*H*-benzo[*d*]imidazole-4-carboxamido)phenyl Ethylmethylcarbamate, RIV–BIMb (**5a**)

This was synthesized from 3-aminophenyl ethylmethylcarbamate (**3a**). The completion of the reaction was controlled by TLC (AcOEt/hexane, 5/1). The crude solid was purified by recrystallization in AcOEt/ethanol, affording the pure compound **5a** as a brown-coloured solid. Yield = 71.4%. M.P. = 222 °C. RF (AcOEt/hexane, 5/1) = 0.74. ^1^H NMR (300 MHz, MeOD-*d*_4_), δ (ppm): 8.21 (d, 1H, *J* = 9 Hz, *Ph*), 7.96 (d, 1H, *J* = 9 Hz, *Ph*), 7.79–7.83 (m, 2H, *Ph*), 7.54 (d, 1H, *J* = 9 Hz, *Ph*), 7.35–7.43 (m, 3H, *Ph*), 7.02–7.06 (m, 2H, *Ph*), 6.91 (d, 1H, *J* = 9 Hz, *Ph*), 3.54 (q, 1H, *J* = 9 Hz, N*CH_2_*CH_3_ rotamer), 3.42 (q, 1H, *J* = 9 Hz, N*CH_2_*CH_3_ rotamer), 3.13 (s, 1.5H, N*CH_3_* rotamer), 3.00 (s, 1.5H, N*CH_3_* rotamer), 1.32 (t, 1.5H, *J* = 9 Hz, CH_2_*CH_3_* rotamer), 1.20 (t, 1.5H, *J* = 9 Hz, CH_2_*CH_3_* rotamer). ^13^C NMR (75 MHz, DMSO-*d*_6_), δ (ppm): 163.66, 156.7, 153.68, 153.57, 151.76, 151.22, 139.88, 132.21, 129.61, 128.65, 122.85, 122.37, 119.82, 117.12, 117.01, 116.18, 114.17, 113.11, 43.59, 34.01, 33.67, 13.16, 12.35. HRMS (ESI) calculated for C_24_H_23_N_4_O_4_ [M + H] 431.1719, found 459.1731.

##### 3-((2-(2-Hydroxyphenyl)-1*H*-benzo[*d*]imidazole-4-carboxamido)methyl) Phenyl Ethylmethylcarbamate RIV–BIMb (**5b**)

This was synthesized from 3-(aminomethyl)phenyl ethylmethylcarbamate (**3b**). The completion of the reaction was controlled by TLC (AcOEt/hexane, 5/1). The crude solid was purified by chromatography column (with eluent AcOEt/hexane, 5/1), affording the pure compound **5b** as a light-yellow-coloured solid. Yield = 38.8%. M.P. = 157 °C. RF (AcOEt/hexane, 5/1) = 0.76. ^1^H NMR (300 MHz, MeOD-*d*_4_), δ (ppm): 8.07 (d, 1H, *J* = 9 Hz, *Ph*), 7.84 (d, 1H, *J* = 9 Hz, *Ph*), 7.73 (d, 1H, *J* = 9 Hz, *Ph*), 7.32–7.40 (m, 2H, *Ph*), 7.20–7.29 (m, 3H, *Ph*), 7.02 (d, 1H, *J* = 6 Hz, *Ph*), 6.90 (d, 1H, *J* = 9 Hz, *Ph*), 6.76 (t, 1H, *J* = 9 Hz, *Ph*), 4.75 (s, 2H, Ph*CH_2_*NH), 3.43 (q, 1H, *J* = 6 Hz, N*CH_2_*CH_3_ rotamer), 3.33 (q, 1H, *J* = 6 Hz, N*CH_2_*CH_3_ rotamer), 3.03 (s, 1.5H, N*CH_3_* rotamer), 2.91 (s, 1.5H, N*CH_3_* rotamer), 1.18 (t, 1.5H, *J* = 6 Hz, CH_2_*CH_3_* rotamer), 1.11 (t, 1.5H, *J* = 6 Hz, CH_2_*CH_3_* rotamer). ^13^C NMR (75 MHz, MeOD-*d*_4_), δ (ppm): 168.78, 163.28, 156.33, 155.52, 153.15, 141.86, 132.84, 130.60, 129.20, 125.61, 123.12, 122.57, 121.90, 121.77, 121.10, 120.03, 118.34, 117.80, 116.04, 45.14, 44.06, 34.52, 34.29, 13.37, 12.61. HRMS (ESI) calculated for C_25_H_25_N_4_O_4_ [M + H] 445.1876, found 445.2189.

##### 4-(3-(2-(2-Hydroxyphenyl)-1*H*-benzo[*d*]imidazole-4-carboxamido) Propyl) Phenyl Ethyl(methyl)carbamate, RIV–BIMb (**5d**)

This was synthesized from 4-(3-aminopropyl)phenyl ethylmethylcarbamate (**3d**). The completion of the reaction was controlled by TLC (AcOEt/hexane, 5/1). The crude solid was purified by chromatography column (with eluent AcOEt/hexane, 5/1), affording the pure compound **5d** as a pale-coloured solid. Yield = 13.1%. M.P. = 126 °C. RF (AcOEt/hexane, 5/1) = 0.69. ^1^H NMR (300 MHz, MeOD-d_4_), δ (ppm): 8.15 (d, 1H, *J* = 9 Hz, *Ph*), 7.81 (d, 1H, *J* = 6 Hz, *Ph*), 7.71 (d, 1H, *J* = 9 Hz, *Ph*), 7.25–7.28 (m, 4H, *Ph*), 6.97 (d, 2H, *J* = 9 Hz, *Ph*), 6.91 (d, 1H, *J* = 6 Hz, *Ph*), 6.76 (t, 1H, *J* = 9 Hz, *Ph*), 3.54 (t, 2H, *J* = 6 Hz, CH_2_*CH_2_*NH), 3.46 (q, 1H, *J* = 6 Hz, N*CH_2_*CH_3_ rotamer), 3.35 (q, 1H, *J* = 6 Hz, N*CH_2_*CH_3_ rotamer), 3.05 (s, 1.5H, N*CH_3_* rotamer), 2.93 (s, 1.5H, N*CH_3_* rotamer), 2.82 (t, 2H, *J* = 6 Hz, Ph*CH_2_*CH_2_), 2.03 (quintet, 2H, *J* = 6 Hz, CH_2_*CH_2_*CH_2_), 1.22 (t, 1.5H, *J* = 6 Hz, CH_2_*CH_3_* rotamer), 1.14 (t, 1.5H, *J* = 6 Hz, CH_2_*CH_3_* rotamer). ^13^C NMR (75 MHz, CD_3_OD-*d*_4_), δ (ppm): 168.94, 155.83, 151.10, 140.70, 140.31, 132.98, 130.51, 129.16, 123.20, 122.87, 122.61, 121.53, 120.36, 118.25, 117.50, 116.93, 116.21, 45.23, 40.23, 33.83, 32.47. HRMS (ESI) calculated for C_27_H_29_N_4_O_4_ [M + H] 473.2189, found 473.2211.

### 2.2. Molecular Modelling

Docking studies of the novel RIV–BIM hybrids with *Human* acetylcholinesterase (hAChE) and *Human* butyrylcholinesterase hBChE) were performed. For this purpose, two X-ray crystallographic structures were downloaded from RCSB Protein Data Bank (PDB entries 4EY7 for the complex of Human acetylcholinesterase (hAChE) with donepezil [46] and 4TPK for the complex of *Human* butyrylcholinesterase (hBChE) with *N*-((1-(2,3-dihydro-1*H*-inden-2-yl)piperidin-3-yl)methyl)-*N*-(2-methoxyethyl)-2-naphthamide [47]). Both crystallographic structures were chosen due to some close similarity between the ligands complexed with the enzymes, and the novel RIV–BIM hybrids. The original ligands, solvent and co-crystallization molecules of the X-ray complex structures were removed using Maestro v.9.3 [48], with the final addition of the hydrogen atoms. Then, the RIV–BIM structures were built in Maestro and, afterwards, to obtain an optimized structure with minimum energy, a random conformational search was performed using Ghemical v.2.0.0 (GPL, Burbank, CA, USA) [49]. Finally, the optimized models of the ligands were docked into the cavity of hAChE and hBChE model structures using GOLD software v.5.2. (CCDC, Cambridge, UK) [50]. Using the ASP as the best fitness function, 100 genetic algorithms steps were performed for the novel ligands to obtain the best matches between the RIV–BIM hybrids and the original ligands of hAChE (radius = 10) and hBChE (radius = 19). Figures of the hypothesized interaction between the ligands and the enzymes were obtained with Chimera software, with the residues between 0 and 5.0 Å from the original position of the ligand in the crystal structure selected as the interest zone. The docking protocol used herein was validated by re-docking the co-crystallized ligands (original ligands) into the corresponding active sites of hAChE or hBChe models [46,47].

### 2.3. Acetylcholinesterase and Butyrylcholinesterase Activity

The inhibition of electric eel acetylcholinesterase (eeAChE) and equine butyrylcholinesterase (eqBChE), both obtained from Sigma-Aldrich (Burlington, MA, USA), was studied using an adaptation of Elmman’s method, as previously described [51]. For this, tris(hydroxymethyl)aminomethane (TRIS) 50 mM buffer at pH = 8.00, 4-(2-hydroxyethyl)-1-piperazine-ethanesulfonic acid (HEPES) 50 mM buffer at pH = 8.00 and HEPES 50 mM buffer at pH = 8.00 with NaCl 50 mM and MgCl_2_ 20 mM were prepared. Stock solutions of 16 mM of acetylthiocholine iodide (AChI) and *S*-butyrylthiocholine iodide (BuChI) in distilled water were prepared and stored in the fridge. Working solutions of AChI and BChI were prepared daily by the proper dilution of the stock solutions in distilled water. A working solution of 5,5′-dithiobis-(2-nitrobenzoic acid) (DTNB) 3 mM in HEPES (NaCl, MgCl_2_) was prepared daily and kept under darkness. Stock solutions of *ee*AChE and *eq*BChE were prepared by dissolving the lyophilized powder enzyme in 10 mL of TRIS buffer under ice and they were divided into aliquots of 50 and 5 UN mL^−1^ that were stored at −20 °C until needed. Stock solutions of 1 mg of each ligand in 1 mL MeOH (with 20% of DMSO) were prepared and working solutions for each ligand were obtained by the corresponding dilution from the stock solutions. A control measurement with 5 replicates (without ligand) and a calibration curve of 5 points with 3 replicates per point was derived. For each solution, fixed volumes of HEPES (374 μL) and *ee*AChE or *eq*BChE (25 μL) and different volumes of ligand (0–50 μL) and MeOH (0–50 μL) were added to quartz cells up to a total volume of 449 μL, and the solutions were left to rest for 15 min immediately after adding the eeAChE or eqBChE. A blank solution containing HEPES (399 μL) and MeOH (50 μL) was prepared at the same time. After 15 min of reaction, AChI or BChI (75 μL) and DTNB (476 μL) were added to both cuvettes and the absorption signal at 405 nm, with a slid width of 1 nm, was recorded over 5 min, with a time interval of 1 s, using a UV–Vis spectrophotometer (PerkinElmer) with the program PerkinElmer UV WinLab. The slope for each calibration point was obtained (absorbance versus reaction time) and the inhibition for each compound was determined using the following equation:

$$\%\;\mathrm{Inhibition}=100-\left(\frac{v_{i}}{v_{0}}\;\times \;100\right)$$

where *v_i_* is the reaction rate (slope) for each point of the calibrate (with ligand) and *v*_0_ is the initial reaction rate (slope) for the control (without ligand). Once the inhibition values of the 5 points of the calibrate and the 3 replicates were obtained, the concentration of each point was plotted versus the percentage inhibition to obtain the inhibitor concentration corresponding to 50% inhibition of the enzyme. The experiments were performed twice.

### 2.4. Inhibition of Aβ_42_ Aggregation

#### 2.4.1. Fluorescence Assays

The inhibition of Aβ_42_ aggregation and Cu(II)-induced Aβ_42_ aggregation, carried out by the RIV–BIM hybrids, was studied using a reported method based on the fluorescence emission of thioflavin T [52,53].

Solutions of phosphate buffer 0.215 M at pH = 8.00, Na_2_CO_3_ 300 μM in distilled water, NaOH 250 mM in distilled water, glycine-NaOH 50 mM buffer at pH = 8.50 and CuCl_2_ 240 μM in phosphate buffer were prepared. Stock solutions of 1 mg L^−1^ of the RIV–BIM compounds were prepared in MeOH (with 20% DMSO). Working solutions of 240 μM of the RIV–BIM hybrids were prepared by the proper dilution of their stock solutions with phosphate buffer. A working solution of glycine-NaOH 50 mM buffer at pH = 8.50 containing 5 μM of thioflavin T (ThT) was prepared daily. Aβ_42_ (GeneCust) was pre-treated with 1,1,1,3,3,3-hexafluoro-2-propanol (HFIP) to obtain a solution of 0.149 mM by brief sonication and vortexing, which was kept covered overnight at room temperature. From the Aβ_42_ solution, aliquots of 250 μL were placed into Eppendorf tubes under ice and set to evaporate inside a hood at room temperature overnight. When the contents of the Eppendorf tubes were dried, the Aβ_42_ aliquots were stored at −20 °C until needed. To obtain the Aβ_42_ working solution, the Aβ_42_ aliquots were dissolved with a freshly prepared mixture of 69.5 μL of CH_3_CN/Na_2_CO_3_ (300 μM)/NaOH (250 mM) (48.3/48.3/3.4, *v*/*v*/*v*) by brief sonication and vortexing to obtain an Aβ_42_ solution of 531.24 μM. To this solution, 392.5 μL of phosphate buffer was added, achieving an Aβ_42_ working solution of 80 μM. To prepare the incubations, three replicates of Aβ_42_ alone (30 μL Aβ_42_ 80 μM + 30 μL phosphate buffer), Aβ_42_ with RIV–BIM (30 μL Aβ_42_ 80 μM + 10 μL RIV–BIM 240 μM + 20 μL phosphate buffer), Aβ_42_ with copper (30 μL Aβ_42_ 80 μM + 10 μL CuCl_2_ 240 μM + 20 μL phosphate buffer) and Aβ_42_ with copper and RIV–BIM (30 μL Aβ_42_ 80 μM + 10 μL CuCl_2_ 240 μM + 10 μL RIV–BIM 240 μM + 10 μL phosphate buffer) were placed in Eppendorfs. To obtain the blank solutions, a similar number of Eppendorfs, replacing Aβ_42_ with phosphate buffer, were prepared. Incubations were performed in a water bath at 37 °C with agitation for 24 h. Afterwards, 180 μL of glycine-NaOH 50 mM buffer at pH = 8.50 containing 5 μM of ThT was added to each Eppendorf and then they were vortexed. Finally, 200 μL of solution from each Eppendorf was placed in the wells of a microplate and fluorescence measurements were monitored using a Spectramax Gemini EM (Molecular Devices) fluorimeter with an excitation wavelength of 446 nm and an emission wavelength of 485 nm. Blank signals were subtracted from the corresponding samples. The fluorescence intensity of the Aβ_42_ + RIV–BIM solution was compared to that of the Aβ_42_ solution to obtain the inhibition of Aβ_42_ self-aggregation for each compound. The fluorescence intensity of the Aβ_42_ + Cu(II) + RIV–BIM solution was compared to that of the Aβ_42_ + Cu(II) solution to obtain the inhibition of the Cu(II)-induced Aβ_42_ aggregation for each hybrid.

#### 2.4.2. TEM Assays

To perform the TEM assays, commercial Aβ_42_ purchased from Sigma-Aldrich was pre-treated with pure HFIP, a beta-sheet disruptor, to obtain homogeneous and monomeric Aβ_42_ [54]. Then, 222 µL of HFIP was added to 1 mg of Aβ_42_, after which the mixture was vortexed and aliquoted into Eppendorfs. The Eppendorfs were placed in a SpeedVac at 45 °C for 30 min until all HFIP was evaporated. Pre-treated Aβ_42_ was stored at −20 °C until use. To start the amyloid incubations, pre-treated Aβ_42_ was equilibrated at room temperature for 30 min. A solution of 5 mM was obtained by dissolution in DMSO and sonication for 5 min. Subsequently, 10 mM HCl was added to obtain a working Aβ_42_ solution of 200 µM. Stock solutions of Cu(II) and RIV–BIM compounds (**4a**–**d**, **5a**,**b** and **5d**) at 200 µM were prepared on the same day and working solutions of Aβ_42_, Aβ_42_ + Cu(II), Aβ_42_ + RIV–BIM and Aβ_42_ + Cu(II) + RIV–BIM (*C*_Aβ42_ = *C*_Cu_ = *C*_L_ = 50 µM) were prepared by diluting stock solutions in 10 mM HCl. Working solutions were incubated for 48 h at 37 °C before being analyzed by TEM. Samples were negatively stained with uranyl acetate 2% (*w*/*v*) and analyzed at 100 kv with a JEOL JEM 1400 Plus transmission electron microscope. Fibrils of Aβ_42_ alone were compared with Aβ_42_ + RIV–BIM fibrils, and fibrils of Aβ_42_ + Cu(II) were compared with Aβ_42_ + Cu(II) + RIV–BIM in order to evaluate the inhibition of the amyloid aggregation, and the widths and lengths of fibrils were measured for each sample. The ImageJ program was used to measure the fibrils (n = 200).

### 2.5. Radical Scavenging Activity

To determine the radical scavenging activity (antioxidant activity) of the novel RIV–BIM hybrids, the DPPH method was performed as previously described in the literature [49]. Working solutions of 20 mg of the ligands were prepared daily in 10 mL of MeOH (with a 10% of DMSO). A working solution of 2 mg DPPH in 100 mL MeOH was prepared daily, and its absorbance was checked to be between 0.75 and 1.00 (a.u.). A calibrate with 5 points and 3 replicates per point was measured, with a fixed volume of DPPH working solution (2.5 mL) and different volumes of ligand (0–1 mL) and MeOH (0–1 mL), up to a final volume of 3.5 mL for each point. Each one of the calibrate points was kept for 30 min under darkness to allow the ligands to exert their antioxidant effects. After 30 min, an auto-zero was performed with MeOH in both sample and reference cells, using a UV–Vis spectrophotometer. Immediately after, the calibrate points were transferred to the quartz cells and their absorbance at 517 nm was measured with a window width of 1 nm using a reference cell with MeOH. The antioxidant activity (%AA) for each point was calculated using the following equation:

$$\%\mathrm{AA}=\frac{A_{DPPH}-A_{ligand}}{A_{DPPH}}\times 100$$

To determine the EC_50_ (i.e., the concentration of the ligand that reduces 50% the DPPH absorbance, %AA = 50%), the %AA and ligand concentration for each point were plotted.

### 2.6. Cell Viability and Neuroprotection of RIV–BIM Compounds in a SH-SY5Y Cell Line

SH-SY5Y human neuroblastoma cells (ATCC-CRL-2266) were kept in culture using Dulbecco’s Modified Eagle Medium (DMEM) (Gibco-Invitrogen, Life Technologies Ltd., Waltham, MA, USA) containing 10% heat-inactivated fetal bovine serum (FBS), 50 U mL^−1^ penicillin and 50 μg mL^−1^ streptomycin. Incubation of the cells occurred at 37 °C in 5% CO_2_. The toxicity of the compounds was tested through cell viability assays, with cells seeded with a density of 0.24 × 10^6^ cells mL^−1^. DMSO was used to dissolve each tested compound (**4a**–**4d**, **5a**, **5b**), affording stock solutions with concentrations of 25 mM. Aliquots of these solutions were stored at −20 °C. Concentrations from 1 μM to 10 μM were screened in order to select the highest non-toxic concentration of each compound. Consequently, compounds **4a**, **4b** and **5b** were tested at 1 μM final concentration; compounds **4c** and **5a** were tested at 2 μM final concentration; compound **4d** was tested at a final concentration of 3 μM. The DMSO concentrations in the culture media were lower than 0.05% (*v*/*v*) and the viability of the SH-SY5Y cells was not altered in a detectable manner.

Pre-incubation of the cells with each compound occurred for 1 h and was followed by incubation with Aβ_42_ or with Fe/Asc for an additional 24 h. A stock solution of Aβ_42_ (Bachem, Torrance, CA, USA) at a concentration of 443 μM was prepared in sterile water and was added to the culture medium at a final concentration of 1 μM. A stock solution of ferrous sulfate (Fe) (Sigma Chemical Co., St. Louis, MO, USA) was freshly prepared in sterile water at a stock concentration of 0.36 M and added to the medium at a final concentration of 2.5 mM. L-Ascorbic acid (Asc) (Sigma Chemical Co, St. Louis, MO, USA) was freshly prepared (80 mM stock solution) and added to the medium at 5 mM final concentration. In all experiments, control conditions were maintained in which Aβ_42_ or Fe/Asc were not added.

The colorimetric MTT (3-(4,5-dimethylthiazol-2-yl)-2,5-diphenyltetrazolium bromide) reduction assay was used to determine cell viability [55]. In this assay, the activity of succinate dehydrogenase, which metabolizes MTT into a formazan that absorbs light at 570 nm, indicates the presence of viable cells. After cell treatments, culture medium was removed and 0.3 mL of MTT (0.5 mg mL^−1^) was added in each well. Subsequently, the plate was incubated at 37 °C for 3 h and the formazan precipitates were solubilized with 0.3 mL of acidic isopropanol (0.04 M HCl/isopropanol). The absorbance of each well was measured at 570 nm. Cell reduction capability was normalized to untreated SH-SY5Y control cells.

All data were expressed as means ± SEMs of at least five independent experiments performed in duplicate. Statistical analyses were performed using one-way ANOVA followed by followed by Dunnett’s test. A *p-*value < 0.05 was considered statistically significant.

### 2.7. Prediction of Pharmacokinetic Properties

In order to determine the drug-likeness properties of the novel RIV–BIM hybrids as anti-AD drugs, predictions of some indicators of their pharmacokinetic profiles were performed in silico. For these, the ligands were built in Maestro and their energy was minimized as described in Section 3.3. Using the program QikProp v.2.5 (Schrödinger, New York, NY, USA) [56], several parameters, such as PSA, o/w partition coefficient, interaction with human albumin, BBB permeability, CNS activity, Caco-2 and MDCK cell permeability, human oral absorption and violations of Lipinski’s rule, were calculated.

### 2.8. Statistical Analysis

In order to simplify the data interpretation, due to the complexity of the results, a chemometric analysis was performed using Statgraphics Centurion XIX. Data were collected from Table 1 and Table 2. Between the multivariant analysis, PCA was chosen to reduce the dimensionality of the data. Listwise elimination and a standardization with a minimum Eigenvalue set as 1.0 were performed.

## 3. Results and Discussion

### 3.1. Molecular Design

The first step towards the design and development of the novel hybrids involved the selection of two different pharmacophoric moieties to endow the final compounds with multiple functionalities so as to guarantee the required multi-targeting activity to fight AD. Therefore, rivastigmine (RIV) was chosen as the first moiety to assure a good inhibition of AChE and BChE. Hydroxyphenylbenzimidazole (BIM) was selected due to its expected ability to bind metals involved in AD, inhibit self- and Cu(II)-induced Aβ_42_ aggregation and reduce ROS production. In addition, variable numbers of methylene groups were included in the linkers between the RIV and BIM moieties (n = 0, 1, 2 and 3) (see Figure 1) in order to tune the desired dual interaction with both active binding sites of the AChE (see Section 2.2). The structural variation associated with the position of the carboxylic acid substituent (BIMa, BIMb) was intended to be used to explore the potential effects of different conjugate spatial conformations (linear (4) and twisted (5) series) on interactions with the target biomolecules and metal ions.

### 3.2. Synthesis of the Compounds

**Scheme 1:**
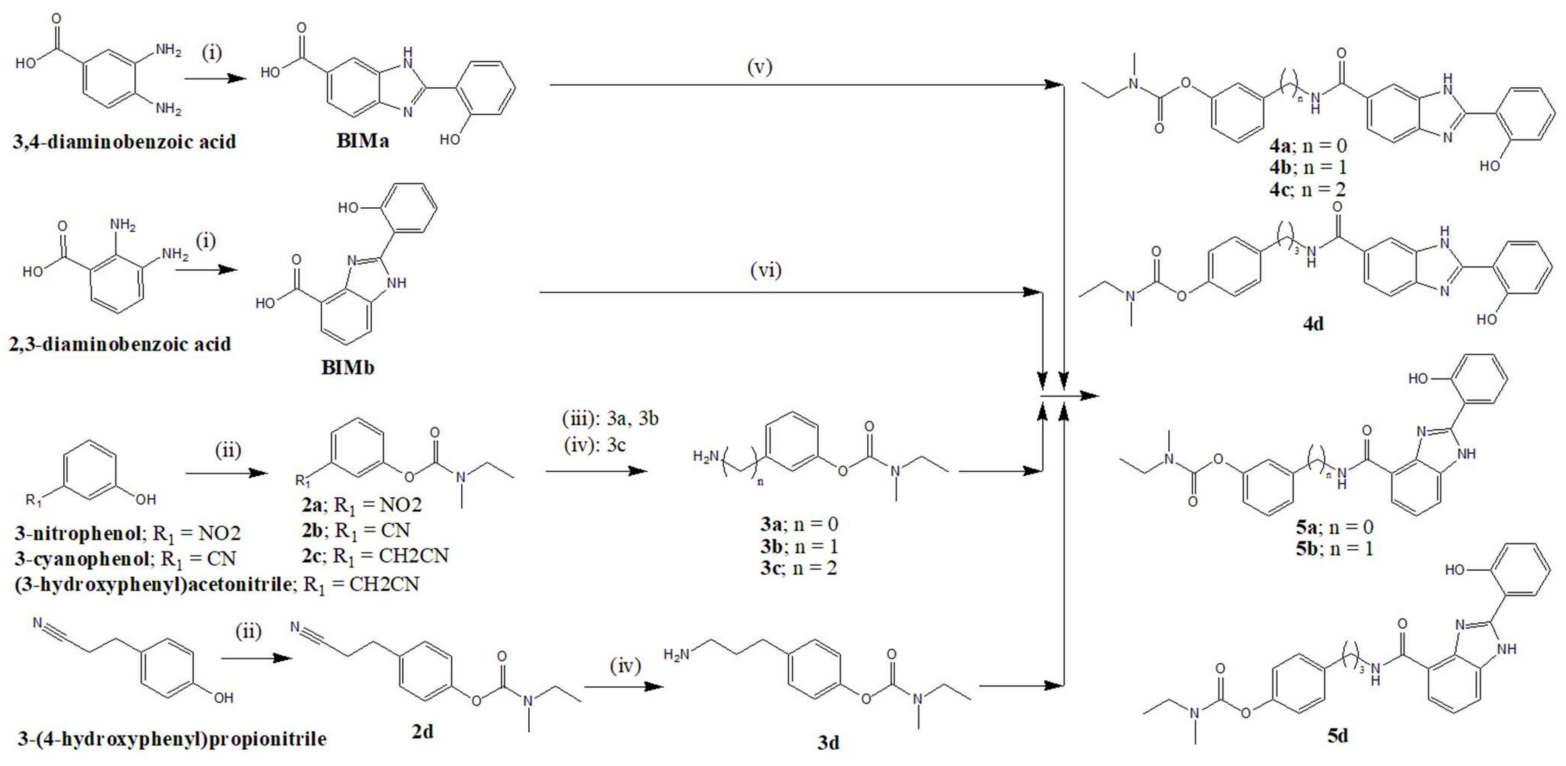
(i) Salicylaldehyde, Na_2_S_2_O_5_, DMA, 100 °C, 12 h; (ii) *N*-ethyl-*N*-methylcarbamoyl chloride, TEA, 95 °C, 12 h; (iii) H_2_, MeOH, 10% Pd-C, 4 h, 4 bar; (iv) H_2_, MeOH/HCl, 10% Pd-C, 6 h, 4 bar; (v) 1: BIMa + NMM, TBTU, dry DMF, water-ice-cooled, N_2_ atm, 50 min; 2: BIMa mixture 1 over **3a**-**d**, N_2_ atm, 20 h; (vi) 1: BIMb + HOBT, EDCI, dry DMF, water-ice-cooled, N_2_ atm, 50 min; 2: BIMb mixture 1 over **3a**, **3b**, **3d**, N_2_ atm, 20 h.

The synthesis of the present set of seven new rivastigmine–hydroxyphenylbenzimidazole hybrids (**RIV–BIMs**; **4a**–**4d** and **5a**, **5b**, **5d**) was performed according to Scheme 1. The intermediate hydroxyphenylbenzimidazole–carboxilic acid derivatives (**BIMa** and **BIMb**) were synthesized according to a previously reported method [35] involving a Mannich reaction between the 3,4- or 3,2-diamino benzoic acid and salicylaldehyde followed by cyclization, in the presence of the reducing agent sodium metabisulfite, in *N*,*N*-dimethylacetamide (DMA) and under high temperature (100 °C), affording the expected compounds with good yields (79.9−86.6%). To obtain the amino (alkyl) rivastigmine derivatives (**3a**–**3d**), a sequential two-step reaction was used. The first step involved the reaction of *N*-ethyl-*N*-methylcarbamoyl chloride with the corresponding nitro- or cyano(alkyl)-phenols by refluxing in dry CH_3_CN and basic medium, triethylamine (TEA), to obtain the corresponding phenylcarbamate intermediates (**2a–2d**) with almost quantitative yields (76.7−99.4%). The following step involved a standard hydrogenolysis of the nitro- or nitrile to the amino groups of these carbamate intermediates, which was carried out in methanol and H_2_ atmosphere (4 atm), though in the case of the alkylnitrile derivatives a catalytic amount of HCl was used, with general yields in the range of 22.0−76.6%. The final step involved the condensation of the carboxylic acid derivatives (**BIMa** and **BIMb**) with the amine-rivastigmine derivatives (**3a–3d**) using standard peptide coupling reagents, namely, 2-(1*H*-benzotriazole-1-yl)-1,1,3,3-tetramethylaminium tetrafluoroborate (TBTU) or 1-ethyl-3-(3-dimethylaminopropyl)carbodiimide (EDCI), in anhydrous DMF under N_2_ atmosphere, affording the corresponding final **RIV–BIM** hybrid compounds (**4a**–**d**, **5a**, **5b**, **5d**) with yields in the range of 23.1−73.9%.

### 3.3. Molecular Modelling

It is well recognized that the overexpression of cholinesterase enzymes results in the degeneration of cholinergic neurons, with deficiency of the neurotransmitter ACh and decline in cognitive functions. Since both AChE and BChE are involved in the blockage of this process, dual cholinesterase inhibition has been explored in the last decade as a new anti-AD drug therapy. To develop effective inhibitors, it is important to understand the structural distinctions between human AChE (hAChE) and BChE (hBChE). Both enzymes are closely related, with an amino acid sequence homology of ca 65%, as well as an active site located at the bottom of the ~20 Ẳ deep hydrophobic gorge, while the peripheral anionic site (PAS) is positioned at the entrance of the gorge [57,58]. The active sites of both enzymes are composed of a catalytic triad, an acyl-binding pocket and a choline binding site. Their main structural differences are the dimensions of their active sites, the active site being wider in BChE due to the replacement of two phenylalanine residues in the acyl binding pocket of AChE with two flexible amino acid residues in BChE [59]. This allows a better accommodation and the binding of bulkier compounds within the active site located at the bottom of the gorge of BChE.

**Figure 2:**
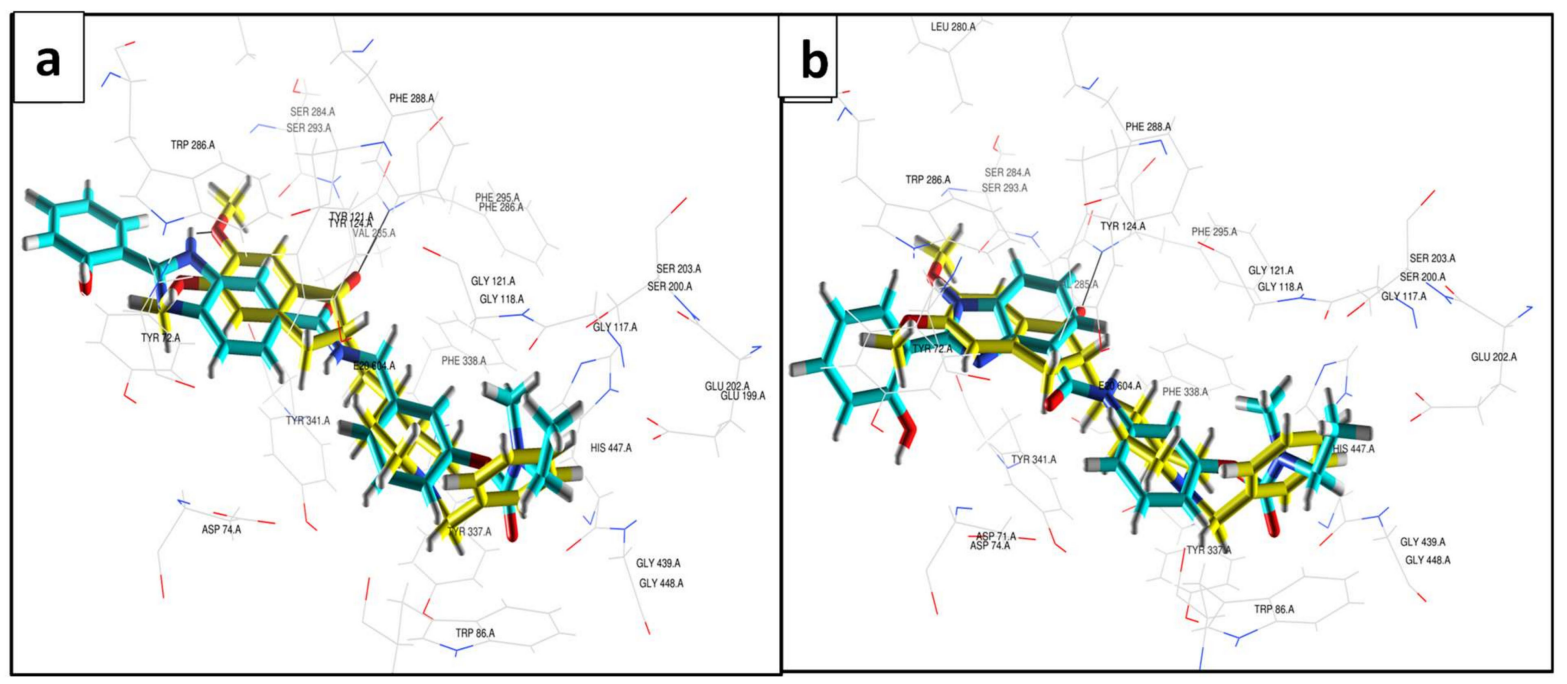
Docking results for RIV–BIM hybrids (blue) within hAChE and a comparison with the original ligand, donepezil (yellow; PDB code: 4EY7 [46]): (**a**) **4b**; (**b**) **5a**.

Our molecular modeling studies involved in silico docking-based virtual screenings of the series of RIV–BIM hybrids inside the active site cavities of hAChE and hBChE. A visualization of the docking poses of the RIV–BIM hybrids inside the hAChE cavity is shown in Figure 2 and Figure S1. A brief global analysis of these modelling results indicates that all the hybrids are well-accommodated inside the enzyme active site and in close proximity to some significant residues of the enzyme [59], such as Phe295, Trp286, Tyr124, Tyr72, Asp74, Tyr337 and Trp86. In particular, Figure 2 illustrates the docking models of hAChE and two representative examples of the series of linear (**4b**) and twisted (**5a**) compounds, showing in both cases a quite similar accommodation and spatial distribution in the hAChE active cavity as compared with the original ligand. Since all the RIV–BIM hybrids have longer structures than the original ligand (donepezil), their accommodation in the enzyme cavity results in the BIM moiety being orientated towards the PAS, while the RIV portion is inside the CAS (compounds **4a**–**4d** and **5a**; see Figure 2 and Figure S1). Moreover, in the case of compound **4b**, an established H-bond between the hydroxyl group of the BIM moiety and Tyr72 can be illustrated. For compounds **5b** and **5d** (in Figure S1), an inversion in the position of the respective moieties inside the cavity of hAChE was detected, which may signify a lower inhibitory potential for these longer compounds of the twisted series. In fact, donepezil is a more potent inhibitor due to the established π–π stacking of the aromatic groups at both ends (benzyl in CAS and indanone in PAS) [46] as well as the hydrogen bond formation between the indanone oxygen atom and Phe295.

**Figure 3:**
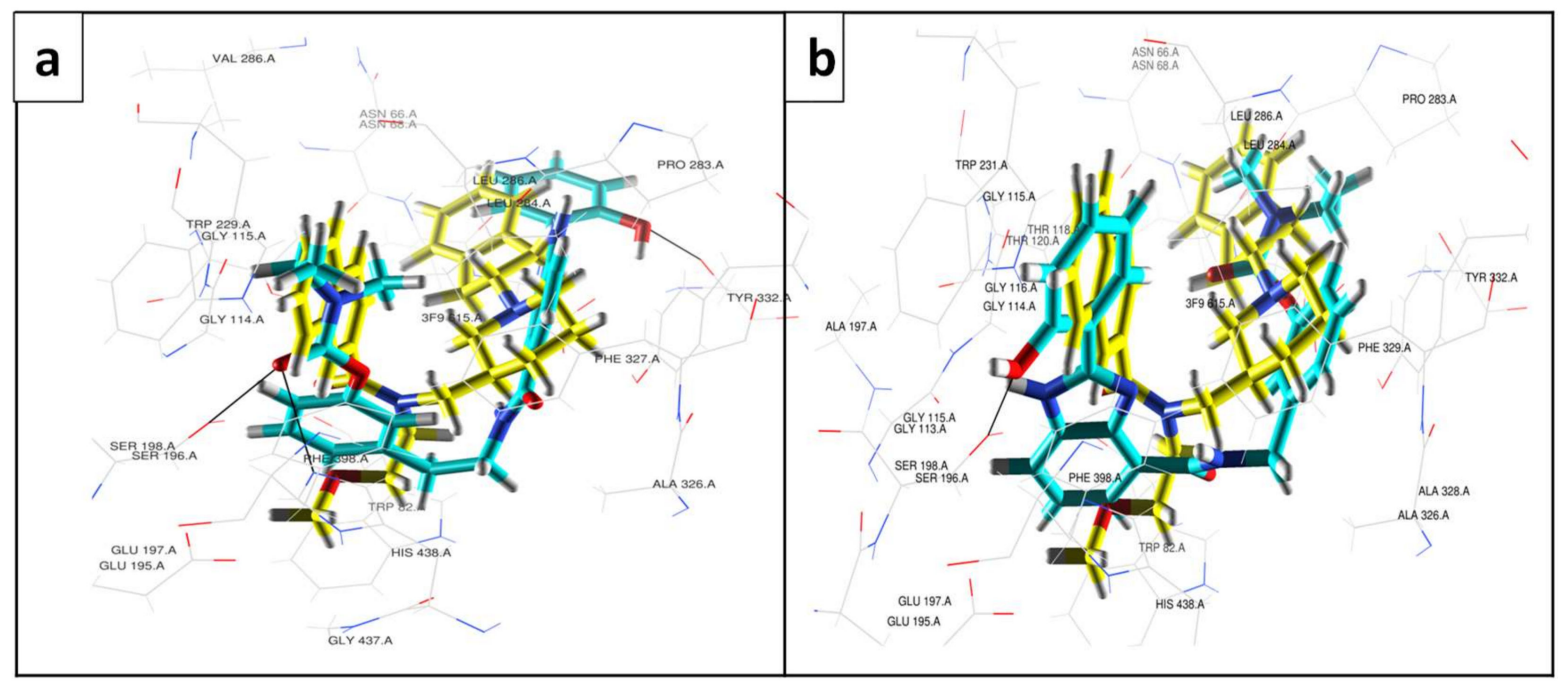
Docking results for RIV–BIM hybrids (blue) within hBChE and a comparison with the original ligand, *N*-((1-(2,3-dihydro-1*H*-inden-2-yl)piperidin-3-yl)methyl)-*N*-(2-methoxyethyl)-2-naphthamide (yellow; PDB code: 4TPK [46]): (**a**) **4c**; (**b**) **5b**.

Regarding human butyrylcholinesterase (hBChE), the docking results depicted in Figure 3 and Figure S2 show that all the RIV–BIM hybrids are well-accommodated inside the enzyme active site, with potential binding interactions with some important active residues of the enzyme [47], such as Tyr332, Trp82, His438, Ser198, Phe398, Trp231, Leu286 and Val288. Figure 3 shows the docking results for two representative examples of the series of linear (**4c**) and twisted compounds (**5b**), revealing them to be well-accommodated in the BChE active cavity, similarly to the original ligand. In the original ligand, the naphthalene moiety fully occupies the acyl binding pocket, establishing π–π stacking interaction with Trp231, while a 1*H*-indene ring is placed over residues Ile69 and Asp70 at the PAS portion. All the linear (**4a**–**4d**) RIV–BIM compounds are longer than the original ligand and those with longer chains have the BIM moiety orientated to the entrance of the gorge, while the RIV portion is accommodated inside the CAS. On the other side, the twisted series (**5a**, **5b** and **5d**) seem to bend better inside the cavity of BChE, orientating the BIM moiety to the CAS and the RIV portion to the PAS.

Overall, the results of the molecular simulations indicate that this class of designed drugs can be good dual inhibitors of the targeted enzymes, and the twisted series eventually appears to be accommodated better in the active site of BChE, encouraging the hypothesization of eventual selective behaviour towards this enzyme.

### 3.4. Enzyme Inhibitory Activity of the Compounds

The inhibition of the *Electrophorus electricus* AChE (*E*eAChE) and the equine BChE (*Eq*BChE) enzymes by the newly synthesized hybrids and some marketed anti-ChE drugs (donepezil, rivastigmine, tacrine) as reference compounds were evaluated employing a previously described method [35]. The results, expressed as IC_50_ values, are shown in Table 1. Regarding AChE inhibition, among the RIV–BIM compounds, **4b** exhibited the best inhibitory capacity value (IC_50_ = 14 μM), in accordance with the better fitting found for **4b** (see Figure 2), followed by **4a**, **5a** and **4c** with identical capacity (IC_50_ = 17.6–17.7 μM), while lower inhibitory capacities were found for compounds **5d**, **4d** and **5b** with IC_50_ values in the range of 21.4–31.7 μM. Interestingly, the results for AChE inhibition do not show any important dependence of activity on linker size, while all the synthesized hybrids presented better AChE inhibition than rivastigmine, proving the improved activity of the RIV–BIM conjugates as compared with the RIV parent molecule. However, as expected, their activity was lower than those of the potent acetylcholinesterase inhibitors donepezil and tacrine. Concerning BChE inhibition, only compound **5b** (IC_50_ = 0.30 μM) showed improvement relative to rivastigmine (IC_50_ = 0.39 μM). Nevertheless, the twisted series (IC_50_ = 0.3–1.7 μM) showed better inhibitory power than the linear one (IC_50_ = 4.8–19.2 μM), with compounds **5a** (0.9 μM) and **5d** (1.7 μM) exhibiting similarly high BChE inhibitory activity in the same range as donepezil (IC_50_ = 1.42 μM). These results are in consonance with molecular modelling studies which revealed that twisted-like compounds (**5a**, **5b**, **5d**) are favoured when it comes to accessing the cavity of BChE, with less good results for longer linker analogues in both series (compounds **4d** and **5d**). The selectivity index (SI), a parameter that describes the different affinity for AChE and BChE, IC_50_(AChE)/IC_50_(BChE), was higher than 1 for all the developed hybrids, indicating a better interaction of the inhibitors with BChE than with AChE.

This can be rationalized by the inherent size and structure of the compounds. In fact, the bigger size of all the developed hybrids, when compared with those of the original ligands (see Section 2.2), seems to allow a better accommodation in the wider active site of BChE, and the twisted structure renders the compounds more flexible, allowing a better interaction with the residues of the enzyme active site.

In fact, to achieve synergistic therapeutic effects, a multi-target molecule should ideally employ balanced activities towards varied targets, with IC_50_ values in vitro within an order of magnitude of each other [60]. Indeed, the compounds developed in this study, except for **5b** (SI (**5b**) = 105.7), obey this rule in terms of cholinesterase’s inhibition and therefore can be considered dual inhibitors.

### 3.5. Inhibition of Self- and Cu(II)-Induced Aβ_42_ Aggregation

The effect of the RIV–BIM compounds on Aβ_42_ aggregation, in the presence and absence of Cu(II), was evaluated using two different techniques: molecular fluorescence spectroscopy and transmission electron microscopy (TEM). The purpose of fluorescence measurements was to quantify the aggregation of amyloid fibrils, while TEM allowed us to observe the fibrils directly and measure their widths under the effects of the compounds. Moreover, due to potential copper-induced fluorescence quenching, TEM assays were also performed as an alternative fluorescence-independent technique, therefore avoiding eventual quantitative errors.

The anti-amyloidogenic capacity of the compounds was assessed in vitro using the thioflavin T (ThT) method [35,52]. To record fluorescence emission, excitation (446 nm) and emission (485 nm) wavelengths were used. When ThT binds to amyloid fibrils, an increase in absorbance and emission of this dye occurs with concomitant red shifts of the respective spectra. The percent of inhibition of Aβ_42_ aggregation is shown in Table 1, and the assays were performed in the presence of 20 μM of inhibitor. These concentration conditions were used to overcome solubility problems of some compounds in the aqueous phosphate buffer working medium. Compounds **4c** and **4d** are very weakly effective in the inhibition of amyloid self- and Cu(II)-induced aggregation, but all the other compounds (**4a**, **4b**, **5a**, **5b** and **5d**) present average/good inhibition capacity regarding Aβ_42_ self-aggregation (39.0–58.7%) These compounds are more potent inhibitors than tacrine (28.1%), though weaker than curcumin (65.7%), a selected reference compound, as an anti-amyloidogenic agent with a 62% Aβ inhibition at 50 μM concentration reported [61]. Moreover, the inhibition of Cu(II)-induced Aβ_42_ aggregation by compounds **4a**, **4b**, **5a**, **5b** and **5d** is also moderate/good (41.2–60.8%), with values only slightly higher than those obtained for self-aggregation inhibition, suggesting that no substantial effect can be observed due to the presence of Cu(II). In fact, the role of Cu(II) in the promotion of Aβ fibril formation is not yet clear, and it can be hypothesized that either Cu(II) chelators are eventually able to compete with the fibrils for Cu(II), therefore reducing aggregation, or that Aβ binding to Cu(II) can lead to some precipitation of amorphous deposits of the peptides rather than the formation of β sheets [62,63].

Regarding the inhibition of Aβ aggregation, the obtained results seem to indicate that, once more, the twisted series (**5**) of hybrids present better and more consistent inhibitory activity than the linear series (**4**) and that the inhibition capacity of these RIV–BIM hybrids may be ultimately related to their different abilities to intercalate between fibrils rather than to their Cu(II) chelating power.

**Figure 4:**
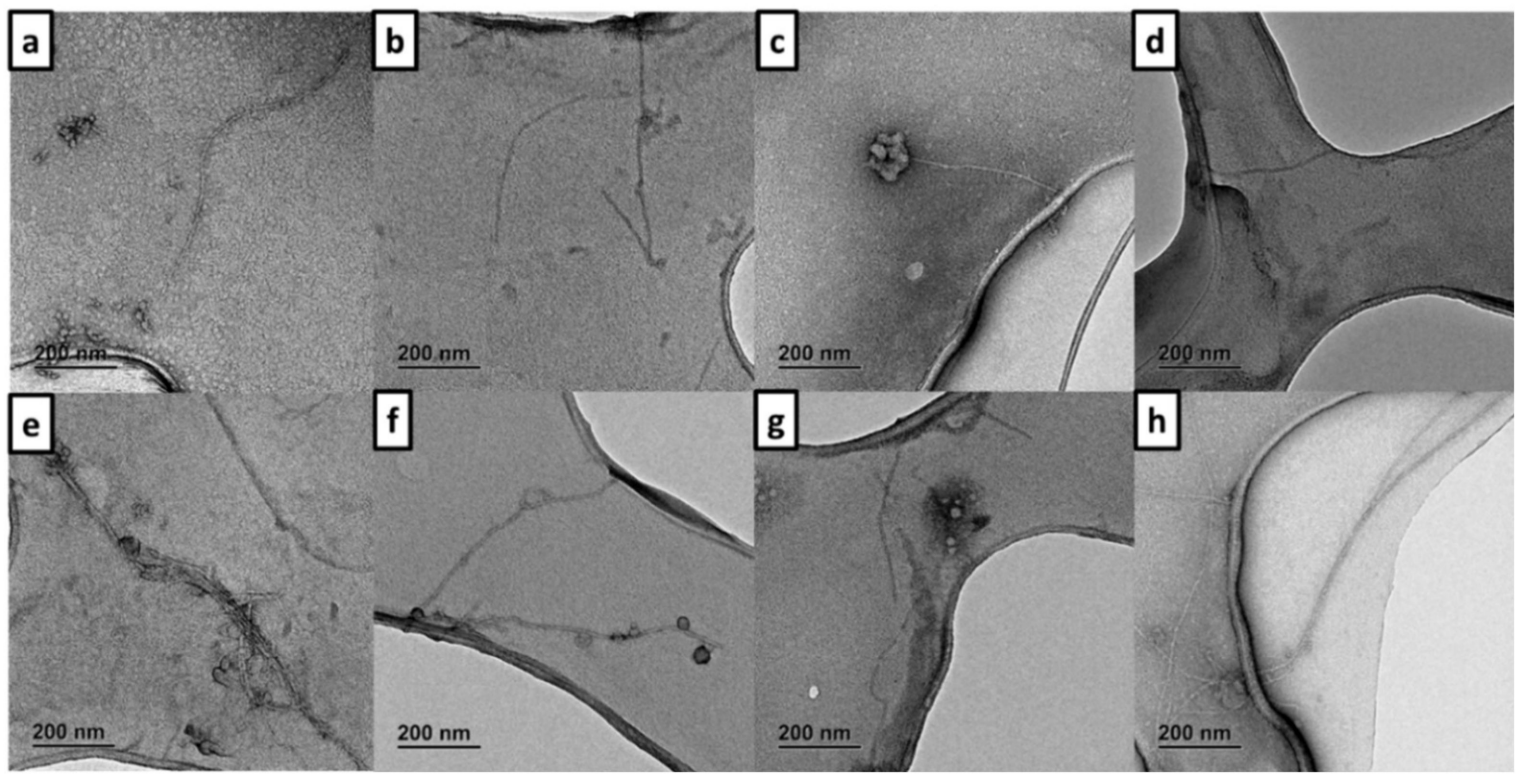
TEM images of Aβ_42_ in the presence of RIV–BIM compounds after incubation at 37 °C for 48 h: (**a**) Aβ_42_ alone; (**b**) Aβ_42_ + **4a**; (**c**) Aβ_42_ + **4b**; (**d**) Aβ_42_ + **4c**; (**e**) Aβ_42_ + **4d**; (**f**) Aβ_42_ + **5a**; (**g**) Aβ_42_ + **5b**; (**h**) Aβ_42_ + **5d**.

To perform the Aβ_42_ aggregation assays by TEM, fibrils were incubated using a fluorescence procedure previously described [20]. The TEM images of Aβ_42_ alone and in the presence of RIV–BIM compounds are presented in Figure 4.

**Figure 5:**
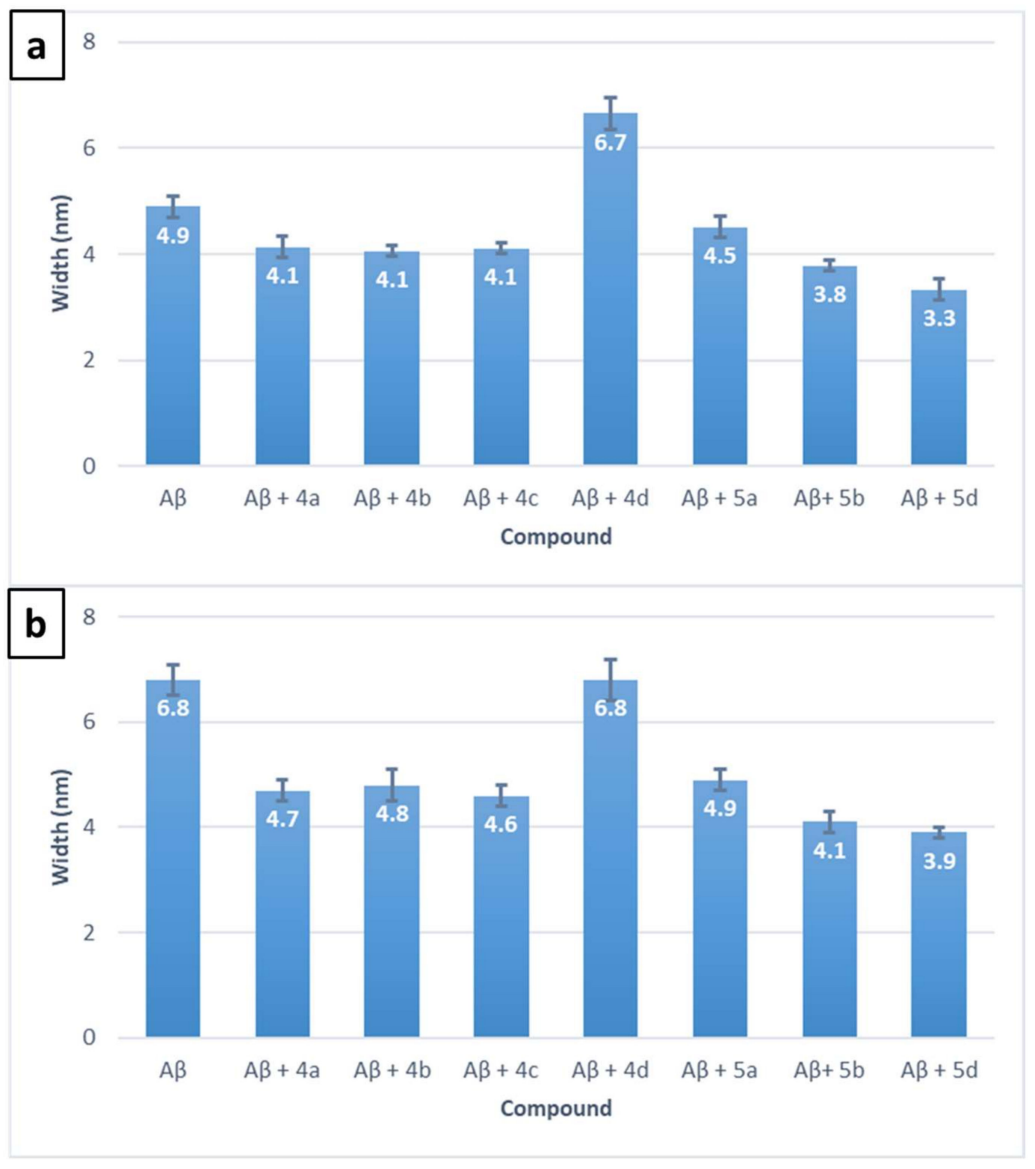
Aβ_42_ fibril widths for TEM incubations measured with ImageJ (±SDs) (N = 200): (**a**) Aβ_42_ + RIV–BIM hybrids in the absence of Cu(II); (**b**) Aβ_42_ + RIV–BIM hybrids in the presence of Cu(II). Statistical analysis was performed using one-way ANOVA and Fisher’s LSD test at 95% significance.

Amyloid fibrils remained disaggregated under the effect of the RIV–BIM compounds after 48 h of incubation. The shortest fibrils were found in the presence of compounds **4a** (Figure 4b), **4b** (Figure 4c) and **5b** (Figure 4g), this last compound having been found to be the best inhibitor of Aβ_42_ aggregation (see Table 1). On the other hand, in the presence of **4c** and **4d** (Figure 4d,e), quite elongated fibrils were present, in accordance with the low values of inhibition of Aβ aggregation previously shown in Table 1 for these compounds. For each of the performed assays, fibril width was measured, and the results are graphed in Figure 5. Aβ_42_ fibril width in the absence of Cu(II) (Figure 5a) was 4.9 ± 0.2 nm, and it was modified in the presence of all RIV–BIM compounds: compound **4d** was the only one that widened amyloid fibrils (6.7 ± 0.3 nm), while the rest of the compounds narrowed them (3.3–4.5 nm). According to results discussed above in this section, compounds **5b** (3.8 ± 0.1 nm) and **5d** (3.3 ± 0.2 nm) reduced fibril width significantly (by 22.4 and 32.7%, respectively), in accordance with their already demonstrated inhibitory capacity in the absence of Cu(II).

**Figure 6:**
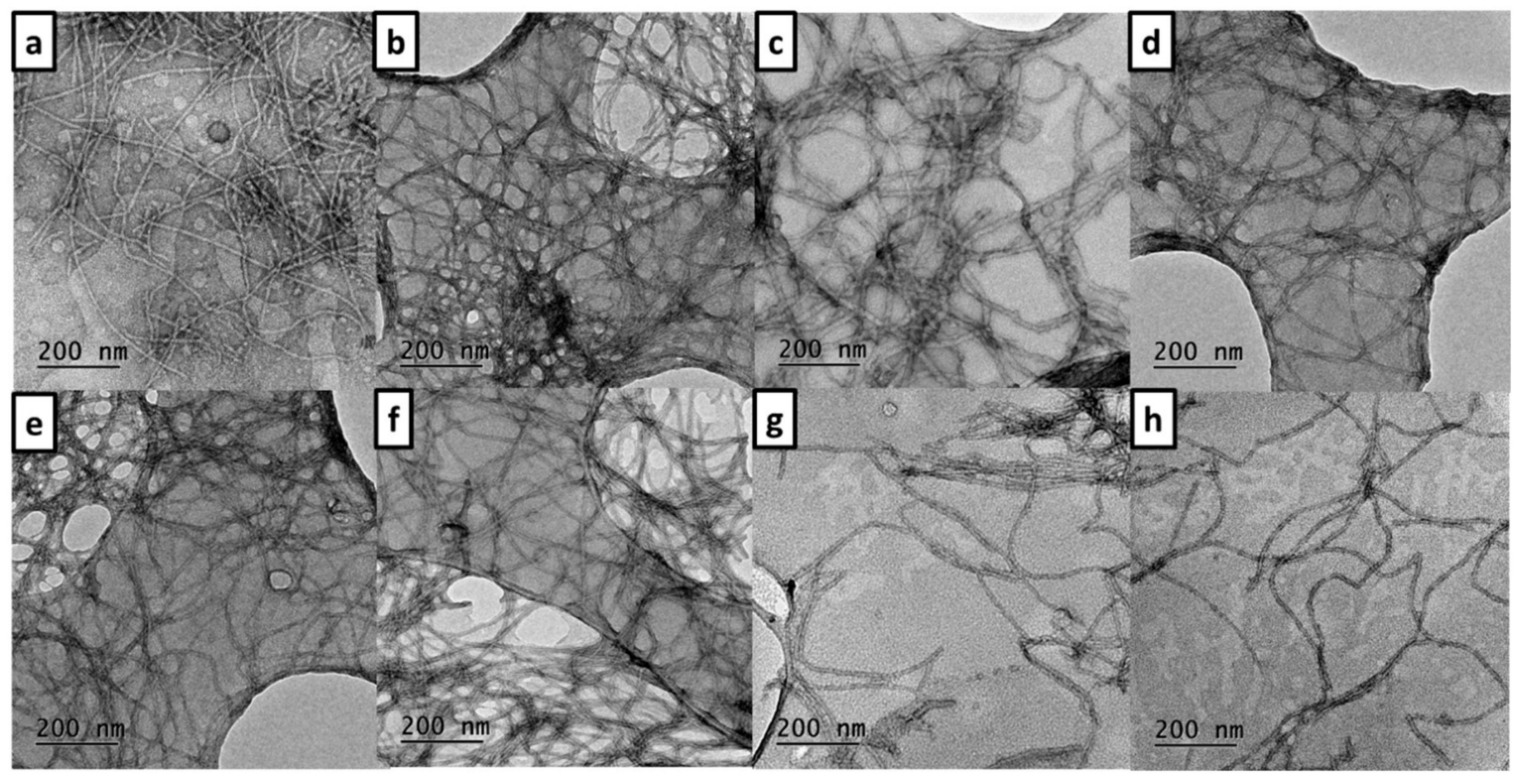
TEM images of Aβ_42_ in the presence of Cu(II) and RIV–BIM compounds after incubation at 37 °C for 48 h (N = 3): (**a**) Aβ_42_ + Cu(II); (**b**) Aβ_42_ + Cu(II) + **4a**; (**c**) Aβ_42_ + Cu(II) + **4b**; (**d**) Aβ_42_ + Cu(II) + **4c**; (**e**) Aβ_42_ + Cu(II) + **4d**; (**f**) Aβ_42_ + Cu(II) + **5a**; (**g**) Aβ_42_ + Cu(II) + **5b**; (**h**) Aβ_42_ + Cu(II) + **5d**.

When fibrils were incubated in the presence of Cu(II) for 48 h (Figure 6), stronger amyloid elongated aggregation was evidenced. In fact, incubation with Cu(II) in the absence of RIV–BIM compounds (Figure 5a) presented higher aggregation than that corresponding to Aβ_42_ alone (Figure 5a). Based on the TEM images contained in Figure 6, compounds belonging to the linear series of hybrids (**4a**–**4d**) seem to exert minor effects on Cu(II)-induced Aβ_42_ aggregation according to the previous fluorescence data. Concerning the twisted series of compounds (**5a**, **5b** and **5d**), the TEM images evidence a sparser amyloid aggregation, mainly for **5b** and **5d**, pointing towards some role of Cu(II) in the aggregation process, although previous fluorescence results did not reveal a substantial effect of copper complexation on Cu(II)-induced Aβ aggregation.

Fibril width for Aβ_42_ + Cu(II) + RIV–BIM incubations was also measured (Figure 5b*)*. When Aβ_42_ was incubated with Cu(II) ((6.8 ± 0.3) nm), the amyloid fibrils suffered a width increase of 38.8%, in contrast to Aβ_42_ alone (Figure 5a). On the other hand, compounds **4a**–**4c** had widths reduced to normal Aβ_42_ levels (4.6–4.8 nm), as did **5a** (4.9 ± 0.2 nm), while **4d** did not undergo any width reduction. Moreover, compounds **5b** and **5d** showed a width reduction that was even greater (39.7 and 42.6%, respectively) in comparison to Aβ_42_ + Cu(II) fibrils.

Therefore, based on the TEM results, combined also with the fluorescence data, compounds **5a**, **5b** and **5d** were revealed as promising candidates for the inhibition of Aβ_42_ self- and Cu(II)-induced aggregation, as well as being able to effect the reduction of the widths of amyloid fibrils.

### 3.6. Radical Scavenging Activity

The radical scavenging activity of the RIV–BIM compounds was determined by a spectrophotometric method involving the 2,2-diphenyl-1-picrylhydrazyl (DPPH) free radical [35,64]. The results obtained, expressed as EC_50_, are shown in Table 1*,* which also includes the value obtained for the reference compound 1,2-dimethyl-3-hydroxy-4-pyridinone (DMHP). Due to the demonstrated low radical scavenging activity of the RIV–BIM hybrids (mM order), only some selected compounds were evaluated. Among the compounds assayed, **4b** (0.9 mM) was the most active, followed by **5a** (8 mM) and **4c** (10 mM), all of them well below the radical scavenging ability of DMHP (0.157 mM).

### 3.7. Cell Viability and Neuroprotection in a SH-SY5Y Cell Line

The neuroprotective effect of RIV–BIM hybrids was tested by exposing neuroblastoma SH-SY5Y cells to Aβ_42_ or ferrous sulphate and *L*-Ascorbic acid (Fe/Asc), as AD-like stressors. A concentration screening was performed to select a non-toxic concentration for each compound (Figure S3). Although several concentrations of the same compound had different non-toxic effects, some failed to prevent Aβ_42_ and Fe/Asc toxicity. Therefore, concentrations were selected according to abilities to increase neuroprotection by maintaining low toxicity levels.

**Figure 7:**
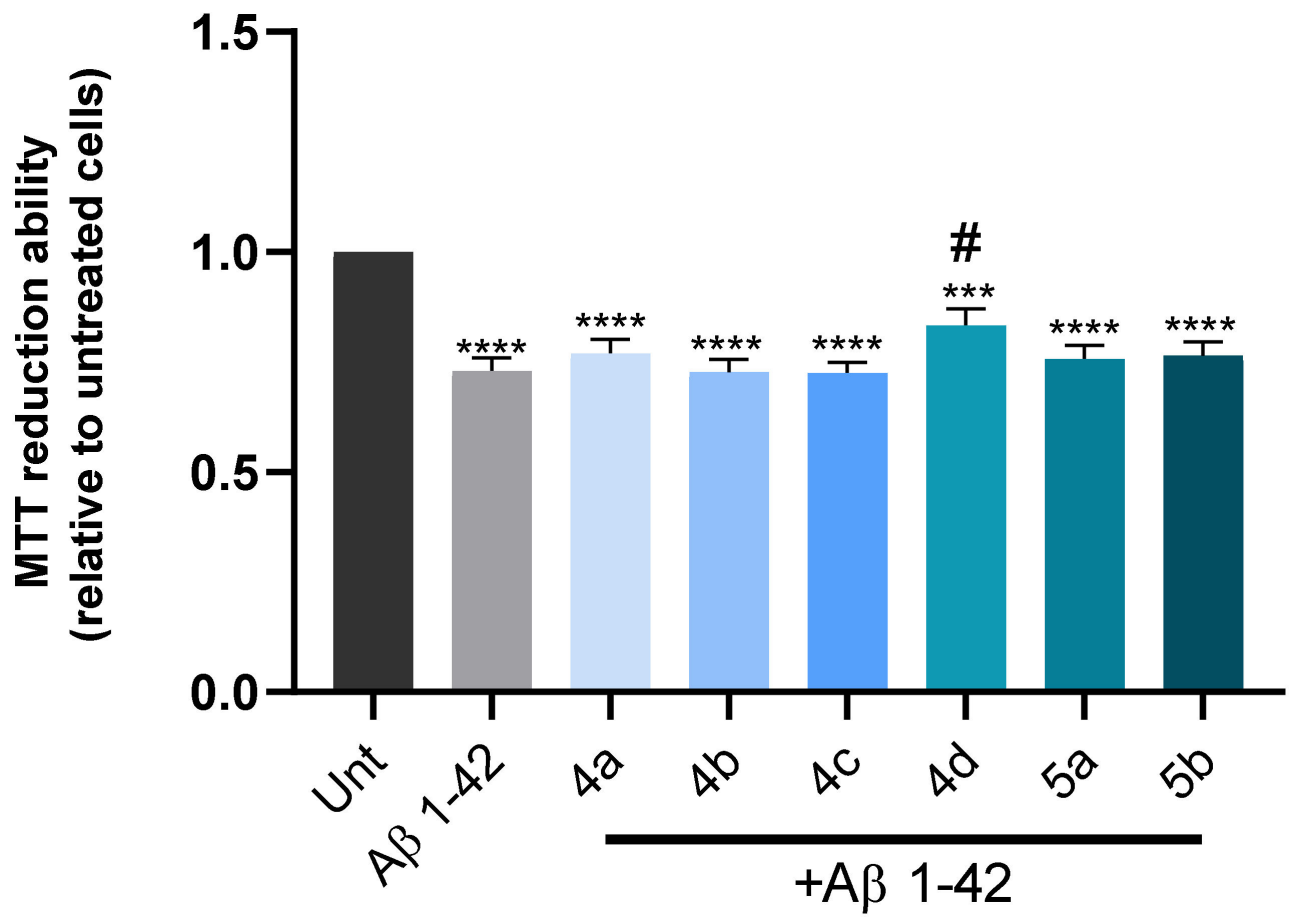
Neuroprotective effects of RIV–BIM compounds against Aβ_42_-induced toxicity in SH-SY5Y cells. Cells were treated with the compounds for 1 h and then Aβ_42_ peptide (1 µM) was added to the medium for 24 h (1 h pre-incubation + 24 h co-incubation). Cell viability evaluation was performed through MTT reduction assay, and the results are expressed relative to those for SH-SY5Y untreated cells, with the means ± SEMs derived from eight different experiments (N = 8). *** *p* < 0.001, **** *p* < 0.0001, significantly different when compared with SH-SY5Y untreated cells; # *p* < 0.05, significantly different when compared with Aβ_42_-treated SH-SY5Y cells. Statistical analysis was performed using one-way ANOVA followed by Dunnett’s test. The concentration used for the compounds **4a**, **4b** and **5b** was 1 µM; 2 µM was used for **4c** and **5a**; and 3 µM for **4d**.

**Figure 8:**
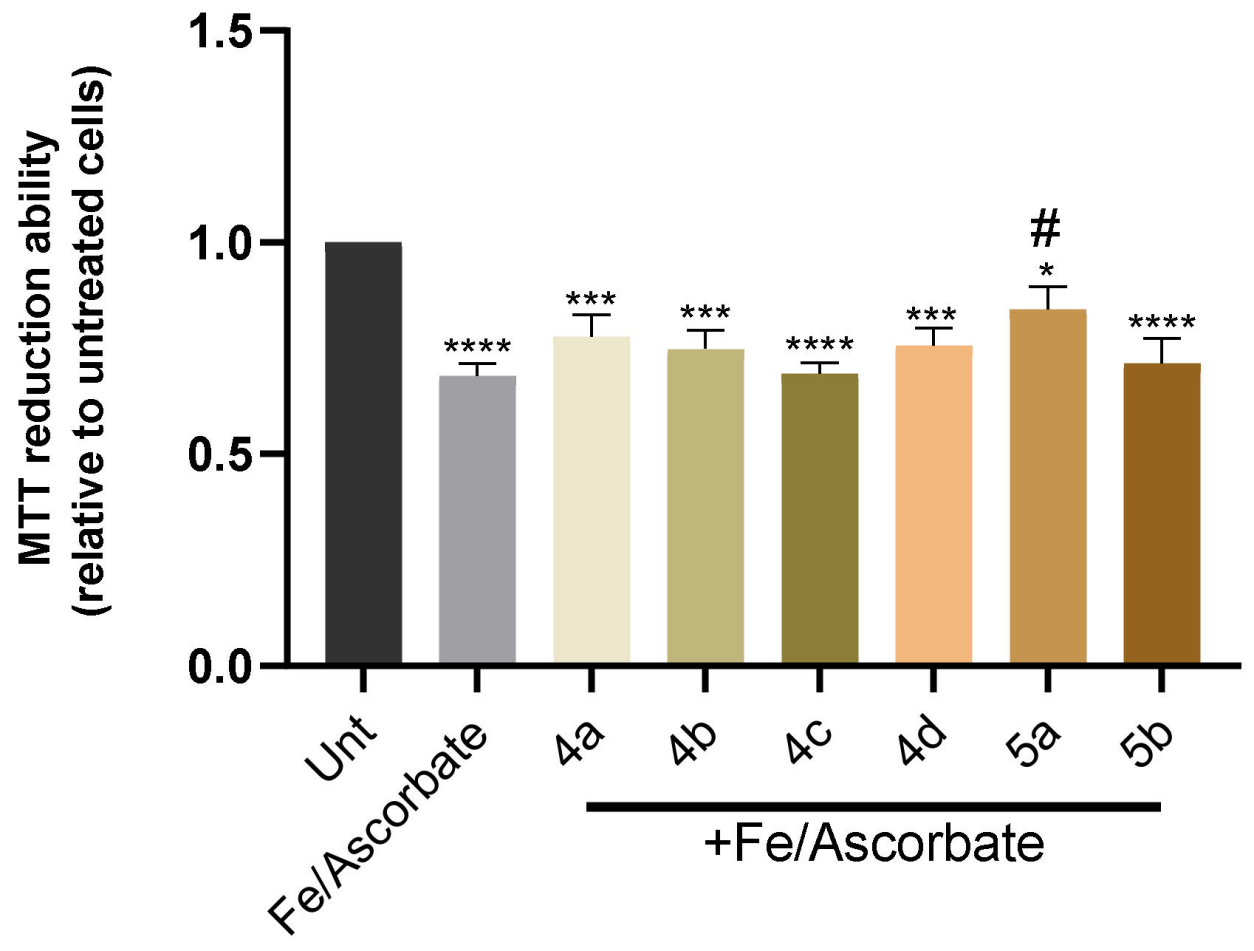
Neuroprotective effects of RIV–BIM compounds against *L*-Ascorbic Acid (AscH(-))/Ferrous Sulphate (Fe) toxicity in SH-SY5Y cells. Cells were treated with the compounds for 1 h and then Fe/Ascorbate (2.5 mM and 5 mM, respectively) was added to the medium for 24 h (1 h pre-incubation + 24 h co-incubation). Cell viability evaluation was performed through MTT reduction assay and results are expressed relative to SH-SY5Y untreated cells, with the means ± SEMs derived from five different experiments (N = 5). * *p* < 0.05, *** *p* < 0.001, **** *p* < 0.0001, significantly different when compared with SH-SY5Y untreated cells; # *p* < 0.05, significantly different when compared with Fe/Ascorbate-treated SH-SY5Y cells. Statistical analysis was performed using one-way ANOVA followed by Dunnett’s test. The concentration used for compounds **4a**, **4b** and **5b** was 1 µM; 2 µM was used for **4c** and **5a**; and 3 µM was used for **4d**.

The increased formation of senile plaques composed of Aβ_42_ peptide aggregates along with the production of reactive oxygen species (ROS) are hallmarks of the neurodegenerative process of AD [13]. Herein, it was observed that Aβ_42_ leads to a 30% decrease in cell viability, and, interestingly, compound **4d** significantly prevented Aβ-induced cell toxicity (Figure 7). Although **4d** revealed moderate/low Aβ inhibition in aqueous media (see Section 2.4, Table 1), some apparent discrepancy with the good results obtained here with cell medium may be explained by the quite different experimental concentration conditions (*C*_inhibitor_ 20 versus 3 μM), suggesting solubility limitations or intermolecular interactions for higher concentration conditions. Additionally, since ROS production is also one of the processes associated with AD [13], to study the neuroprotection of RIV–BIM compounds against oxidative stress, SH-SY5Y cells were also treated with Fe/Asc, which induced a decrease of 30% in the cell viability when compared with untreated cells (Figure 8). In this case, only compound **5a** presented a statistically significant neuroprotective effect by preventing ROS production.

This set of results suggests that compound **5a** (and **4d**) may have a potential therapeutic effect on AD, since it was shown to prevent cell toxicity induced by Aβ_42_ or oxidative stress.

### 3.8. Predicted Pharmacokinetic Properties

To estimate the drug-like behaviour of the RIV–BIM compounds, some pharmacokinetic properties were predicted using the QikProp v.2.5. program [56] and compared with those of the parent drug rivastigmine (RIV) (Table 2). All the hybrids presented an acceptable range of values for molecular weight, PSA (Van der Waals surface area of polar nitrogen and oxygen atoms), octanol/water partition coefficient (except compound **5d**), serum protein binding and brain–blood barrier permeability. Caco-2 cell permeability was high for compounds **5a**, **5b** and **5d** (>500 nm s^−1^) and was in the normal range for the rest of the molecules. Moreover, all hybrids presented acceptable MDCK permeability values and were demonstrated to be easily absorbed through the oral route (oral absorption >80%; see Table 2). The number of violations of Lipinski’s rule of five was 0 for all the compounds, except **5d** (1 violation, clog *P*_o/w_ > 5), which indicates that all of them seem to be suitable as potential oral drug candidates. Compared with the original drug rivastigmine, the insertion of a BIM moiety in compounds of series 4 and 5 is apparently responsible for some lowering of predicted Caco-2 and MDCK cell permeabilities.

Overall, these studies predicted for the compounds generally good pharmacokinetic (PK) descriptors and drug-likeness, including potential good permeability for BBB and other important gut wall membranes as well as oral absorption capacity. However, further future studies will be necessary to complete the PK assessment of ADMET properties, as will experimental results to support the compounds’ potential BBB permeability and to study their metabolism and elimination stages.

### 3.9. Chemometric Analysis

**Figure 9:**
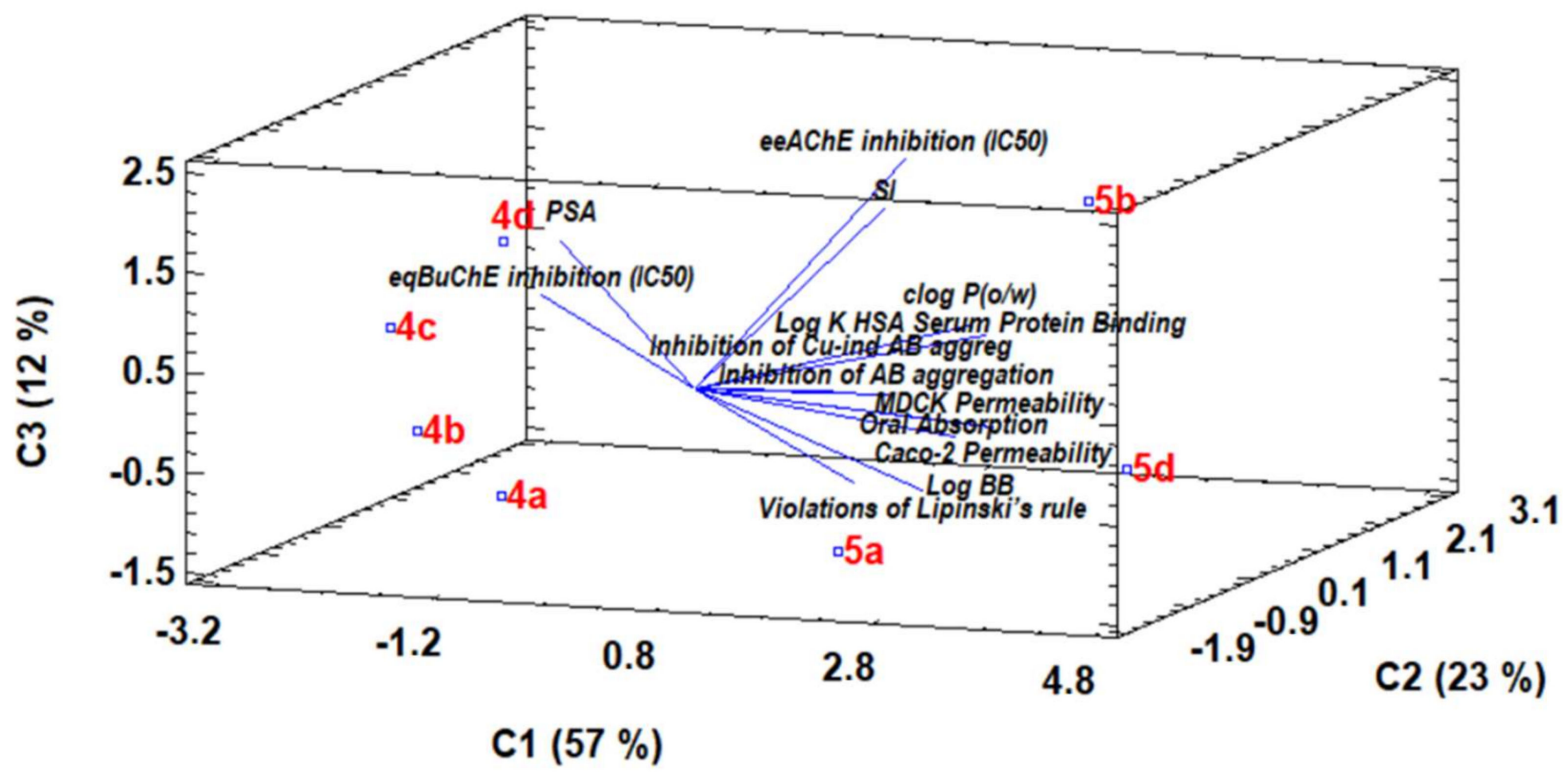
Three-dimensional bigraphic of a dispersion diagram and component weights obtained through a PCA analysis using chemometric tools.

Due to the elevated RIV–BIM data analysed in this work, a principal component analysis (PCA) using chemometric tools was performed. This statistical analysis allowed a more precise differentiation between the RIV–BIM compounds with respect to their properties. The data contained in Table 1 (*ee*AChE and *eq*BChE inhibition (IC_50_), SI, inhibition of Aβ_42_ and Cu(II)-induced Aβ_42_ aggregation) and Table 2 (PSA, clog *P*(o/w), log *K* HSA serum protein binding, log BB, Caco-2 and MDCK permeability, oral absorption and violations of Lipinkski’s rule) were analysed. The PCA analysis with three components was able to explain 91.9% of the variability, demonstrating its high reliability. The 3D bigraphic diagram between the dispersion diagram and component weights (Figure 9) shows that both series of compounds (**4** and **5**) are in opposite sides of the box, indicating that they do indeed have different properties based on their structure regarding the first principal component. The first principal component is explained mainly by oral absorption, Caco-2 and MDCK permeability, clog *P*(o/w), log *K* HSA serum protein binding, inhibition of Aβ_42_ and Cu(II)-induced aggregation, evidencing a relationship between each of these, this being common for compound series **5**. Therefore, these parameters represent the most important differences between series **4** and **5**. Furthermore, between compounds of the same series, there is also a pattern from **a** to **d**. This is more evident in series **4** because of the Lipinski’s rule violation of compound **5d**, which had a high weight in the third principal component. The bigraphic indicates that the IC_50_ of *eq*BChE inhibition increases in the left side of the box, the compounds **4a**–**d** being the ones with the worst values (higher IC_50_ values). On the other hand, the higher IC_50_ of *ee*AChE inhibition generally resulted in higher SI.

## 4. Conclusions

Seven novel RIV–BIM hybrids based on the conjugation of the drug rivastigmine with hydroxyphenylbenzimidazole moieties were designed, prepared and evaluated for their biological properties as potential multi-target anti-Alzheimer´s disease (AD) drugs. Molecular docking modelling showed possible interactions between the RIV–BIM hybrids and the cholinesterases (AChE and BChE), indicating that all the compounds could be well accommodated inside the cholinesterase active site gorges but with dependences of inhibitory activity/selectivity on ligand structural differences. Pharmacokinetic parameters of the compounds were also evaluated *in silico*, predicting adequate drug-likeness properties and potential oral bioavailability. Regarding biological properties, all the compounds revealed higher AChE inhibitory activity than rivastigmine (IC_50_ = 32.1 µM), while compounds of series **5** displayed better inhibition of BChE than those of series **4**, **5b** (IC_50_ = 0.30 µM) and **5a** (IC_50_ = 0.9 µM) in particular. Although all the compounds showed good ChE inhibitory capacity, generally, they presented selectivity for the inhibition of BChE over AChE. Concerning the inhibition of Aβ_42_ aggregation, fluorescence studies demonstrated that series **5** has higher inhibitory activity than series **4**. TEM observations of the effect of RIV–BIM hybrids in amyloid fibril aggregation, in the absence of Cu(II), showed that, generally, the compounds promote fibril narrowing (4.9 nm width in the absence of compounds), with the highest width reduction achieved by **5b** (3.8 nm) and **5d** (3.3 nm). Regarding Cu(II)-induced Aβ_42_ aggregation, TEM assays indicated more intense aggregation in the presence of the metal ion than in its absence, along with a 38.8% increase in fibril width. Compounds **5b** and **5d** were responsible for the highest fibril width reduction in comparison with Aβ_42_ + Cu(II) fibrils. Therefore, TEM studies seem to point towards the role of copper complexation in the inhibition of Aβ_42_ aggregation, although such an effect was not much evidenced by the fluorescence studies. The effects of the compounds in terms of the reduction of toxicity induced by Aβ and ROS in neuronal cells (SH-SY5Y) were also evaluated, and **4d** and **5a,** respectively, showed the best neuroprotective capacities. A principal component analysis (PCA) of the experimental dataset allowed us to group and correlate the new compounds according to the evaluated parameters.

Overall, the novel RIV–BIM hybrids presented here show interesting properties as potential anti-AD drugs, some of them bearing diverse and important anti-AD hallmarks (inhibition of cholinesterases, inhibition of amyloid self-aggregation and Cu(II)-induced aggregation, as well as reduction of ROS and Aβ_42_ toxicity in neuronal cells). Therefore, further studies on this RIV–BIM hybrid family are encouraged, with future developments of these compounds as multi-target anti-AD— and eventually also as anti-Parkinson’s disease (PD)—drug candidates envisaged, since cholinergic transmission disruption and related cognitive decline perturbations have been associated with PD pathology, besides other commonalities and correlated onsets between AD and PD.

## Figures and Tables

**Table 1:** Biological properties of the RIV–BIM hybrids under study as well as those of the comparison compounds.

Comp	Antioxid. ^a^ EC_50_ (mM)	AChE Inhib ^b^ IC_50_ (μM)	BChE Inhib ^b^ IC_50_ (μM)	SI ^c^	Aβ_42_ Self-Aggreg. Inhib ^d^ (%)	Aβ_42_ Cu-Ind. Aggreg. Inhib ^d^ (%)
**4a**	–	17.6 ± 0.6	5.85 ± 0.03	3.03	39.0	41.2
**4b**	0.9 ± 0.1	14 ± 2	6.5 ± 0.4	2.17	39.6	48.8
**4c**	10.6 ± 0.3	17.7 ± 0.8	4.8 ± 0.1	3.69	21.2	22
**4d**	–	24 ± 1	19.2 ± 0.7	1.25	20.1	21.3
**5a**	8 ± 1	17.6 ± 0.4	0.9 ± 0.1	19.6	44.5	45.4
**5b**	–	31.7 ± 0.1	0.30 ± 0.01	105.7	58.7	60.8
**5d**	–	21.4 ± 0.4	1.7 ± 0.2	12.6	42.1	40.3
DMHP	0.157 ± 0.008	–	–	–	–	–
Tacrine	–	0.024 ± 0.007	–	–	28.1	–
Curcumin	–	–	–	–	65.7	62.7
Donepezil	–	0.0075	1.42	0.005	–	–
Rivastigmine	–	32.1	0.39	82.3	–	–

^a^ Radical scavenging activity (DPPH method). ^b^ The values are means of five independent experiments ± SDs. Assays with *ee*AChE and *eq*BChE. ^c^ Selectivity index = IC_50_ (AChE)/IC_50_ (BChE). ^d^ Percent of inhibition of Aβ_42_ aggregation in the presence or absence of copper (40 μM) and 20 μM inhibitor (thioflavin-T fluorescence method). The values are the means of two independent measurements in duplicate (SEM < 10%).

**Table 2:** Predicted pharmacokinetic properties of the RIV–BIM hybrids and the parent RIV drug, obtained using the software QikProp v.2.5 [56].

Molecule	Mol. Weight ^a^	PSA ^b^	clog P^o/w c^	log *K* (HSA) Serum Protein Binding ^d^	log BB ^e^	Caco-2 Permeability (nm s^−1^) ^f^	MDCK Permeability (nm s^−1^) ^g^	Oral Absorption ^h^
**4a**	430.462	114.416	3.884	0.519	−1.449	401	184	91
**4b**	444.489	116.724	4.045	0.563	−1.58	365	166	89
**4c**	458.516	117.689	4.412	0.679	−1.758	333	151	88
**4d**	472.543	116.719	4.695	0.743	−1.724	382	175	89
**5a**	430.462	113.662	4.318	0.673	−1.238	535	251	94
**5b**	444.489	115.249	4.737	0.803	−1.345	539	253	94
**5d**	472.543	115.284	5.398	0.998	−1.5	548	258	93
**RIV**	250.340	40.321	2.488	−0.133	0.475	1381	776	100

^a^ Molecular weight (acceptable range: from 130 to 725). ^b^ PSA (Van der Waals surface area of polar nitrogen and oxygen atoms) (acceptable range: from 7 to 200). ^c^ Predicted octanol/water partition coefficient log *P* (acceptable range: from −2.0 to 6.5). ^d^ Interaction with human albumin (acceptable range: from −1.5 to +1.5). ^e^ Brain–blood barrier permeability (acceptable range: from −3.0 to +1.2). ^f^ Predicted Caco-2 cell permeability in nm/s (acceptable range: 25 is poor and >500 is excellent). ^g^ Predicted MDCK cell permeability in nm/s (acceptable range: <25 is poor and >500 is excellent). ^h^ Percentage of human oral absorption (acceptable range: <25% is poor and >80% is excellent).

## Data Availability

Data is contained within the article and supplementary files.

## Supplementary Materials

The following is available online: https://www.mdpi.com/article/10.3390/biomedicines10071510/s1. Figure S1. Docking results for RIV-BIM hybrids (blue) with hAChE and pose comparisons with the original ligand (yellow, PDB code 4EY7): (a) **4a**; (b) **4c**; (c) **4d**; (d) **5d**. Figure S2. Docking results for RIV-BIM hybrids (blue) with hBuChE and pose comparisons with the original ligand (yellow, PDB code 4TPK): (a) **4a**; (b) **4b**; (c) **5a**; (d) **5d**. Figure S3. Dose-response screening of RIV-BIM compounds in SH-SY5Y cell line to select a non-toxic concentration. Cells were treated with different concentrations of the mentioned compounds for 24 h. To evaluate cell viability, MTT reduction assay was performed. Results are expressed relatively to SH-SY5Y untreated cells, with the mean ± SEM derived from eight different experiments. * *p* < 0.05; ** *p* < 0.01; *** *p* < 0.001; **** *p* < 0.0001, significantly different when compared with SH-SY5Y untreated cells.

## Abbreviations

ACh	Acetylthiocholine
AChI	Acetylthiocholine iodide
AD	Alzheimer’s Disease
ADMET	Absorption, Distribution, Metabolism, Elimination, Toxicity
APP	Amyloid Precursor Protein
Aβ_42_	Beta-amyloid protein, fragment 1-42
BBB	Blood–brain Barrier
BIM	Hydroxyphenylbenzimidazole acid
BCh	Butyrylthiocholine
BChI	S-butyrylthiocholine iodide
Caco	Human colon carcinoma cell line
CNS	Central nervous system
DMA	*N*,*N*-dimethylacetamide
DMEM	Dulbecco’s Modified Eagle Medium
DMF	Dimethylformamide
DMHP	1,2-Dimethyl-3-hydroxy-4-pyridinone
DMSO	Dimethyl sulfoxide
DPPH	2,2-diphenyl-1-picrylhydrazyl free radical
DTNB	5,5′-dithiobis-(2-nitrobenzoic acid)
EC_50_	Half maximal effective concentration
EDCI	1-ethyl-3-(3-dimethylaminopropyl)carbodiimide
*ee*AChE	Electric eel acetylcholinesterase
*eq*BChE	Equine butyrylcholinesterase
FBS	Fetal bovine serum
HD	Huntington’s disease
HEPES	4-(2-hydroxyethyl)-1-piperazine-ethanesulfonic acid
HFIP	1,1,1,3,3,3-Hexafluoro-2-propanol
HSA	Human serum albumin
IC_50_	Half maximal inhibitory concentration
LC	Liquid chromatography
MDCK	Madin–Darby canine kidney cell line
M.P	Melting points
MS-ESI	Electrospray ionization mass spectrometry
MTT	3-(4,5-dimethylthiazol-2-yl)-2,5-diphenyltetrazolium bromide
NMM	*N*-Methylmorpholine
NMR	Nuclear magnetic resonance
PCA	Principal component analysis
PD	Parkinson’s disease
PSA	Polar surface area (Van der Waals surface area of polar nitrogen and oxygen atoms)
RF	Retention factor
RIV	Rivastigmine
ROS	Reactive oxygen species
SEM	Standard error of the mean
SD	Standard deviation
SI	Selectivity index (IC_50_(AChE)/IC_50_(BChE)
TBTU	2-(1*H*-benzotriazole-1-yl)-1,1,3,3-tetramethylaminium tetrafluoroborate
TEA	Triethylamine
TEM	Transmission electron microscopy
ThT	Thioflavin T
TLC	Thin-layer chromatography
TMS	Tetramethylsilane
TRIS	Tris(hydroxymethyl)aminomethane
UV-Vis	Ultraviolet–visible spectroscopy
