# Supplementary material for: Novel Rivastigmine Derivatives as Promising Multi-Target Compounds for Potential Treatment of Alzheimer’s Disease
Source: Biomedicines. 2022 Jun 26;10(7):1510. doi: 10.3390/biomedicines10071510 (PMC9313321; doi:10.3390/biomedicines10071510)
Supplement: Supplementary file 1 [file biomedicines-10-01510-s001.zip › biomedicines-1770151-supplementary.pdf]

Supplementary Information

NOVEL RIVASTIGMINE DERIVATIVES AS PROMISING MULTI-TARGET COMPOUNDS FOR POTENTIAL TREATMENT OF ALZHEIMER'S DISEASE

David Vicente-Zurdo[a,b], Noelia Rosales-Conrado[b], M. Eugenia León-González[b], Leonardo Brunetti[a,c], Luca Piemontese[c], A. Raquel Pereira-Santos[d], Sandra M. Cardoso[d,e], Yolanda Madrid[b], Sílvia Chaves[a,*], M. Amélia Santos[a,*]

[a] Centro de Química Estrutural, Institute of Molecular Sciences, Departamento de Engenharia Química, Instituto Superior Técnico, Universidade de Lisboa, Av. Rovisco Pais 1, 1049-001, Lisboa, Portugal; [b] Department of Analytical Chemistry, Faculty of Chemistry, Complutense University of Madrid, Avenida Complutense s/n, 28040, Madrid, Spain; c) Department of Pharmacy-Pharmaceutical Sciences, University of Bari Aldo Moro, via E. Orabona 4, 70125 Bari, Italy; [d] CNC-Center for Neuroscience and Cell Biology, University of Coimbra, Coimbra, Portugal; [e] FMUC-Faculty of Medicine, University of Coimbra, Coimbra, Portugal.

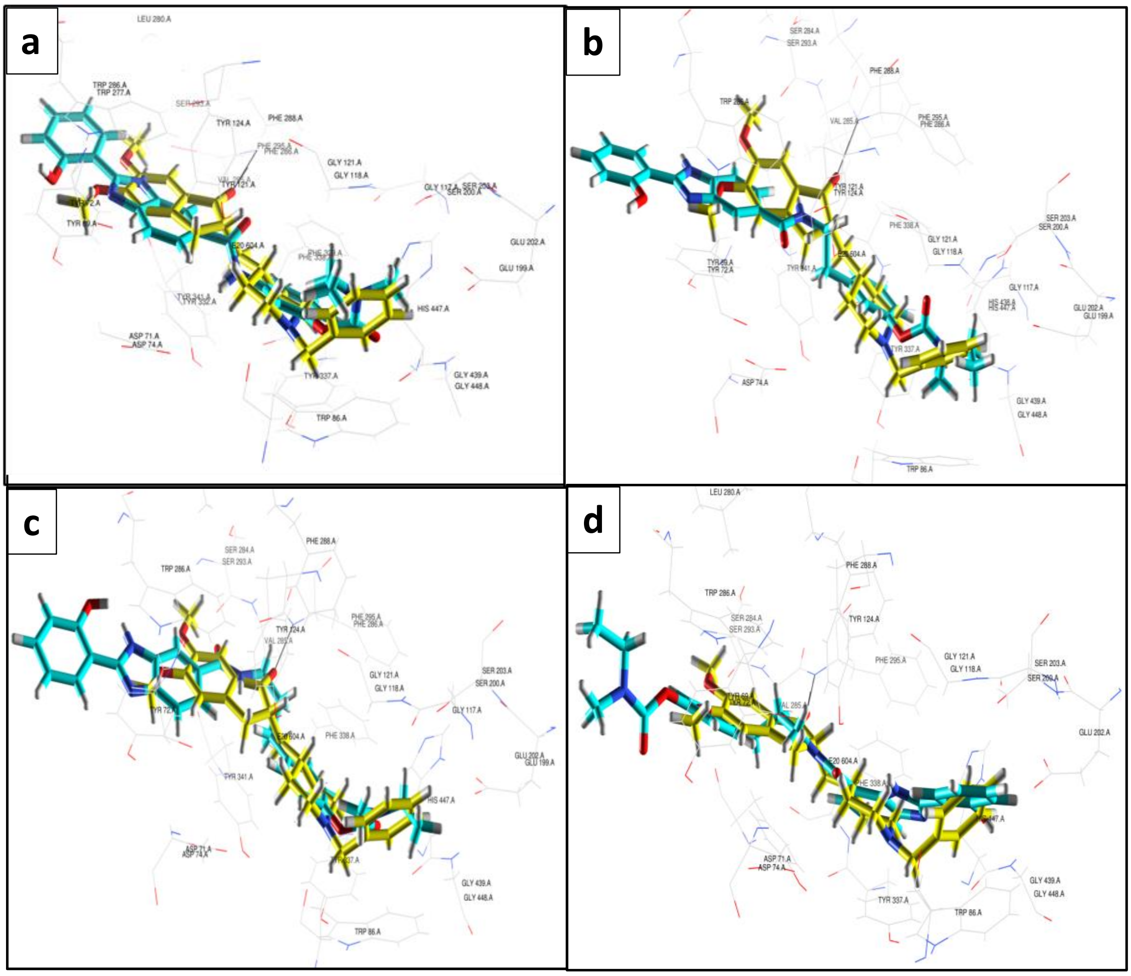

Figure S1. Docking results for RIV-BIM hybrids (blue) with hAChE and pose comparisons with the original ligand (yellow, PDB code 4EY7): (a) **4a**; (b) **4c**; (c) **4d**; (d) **5d**.

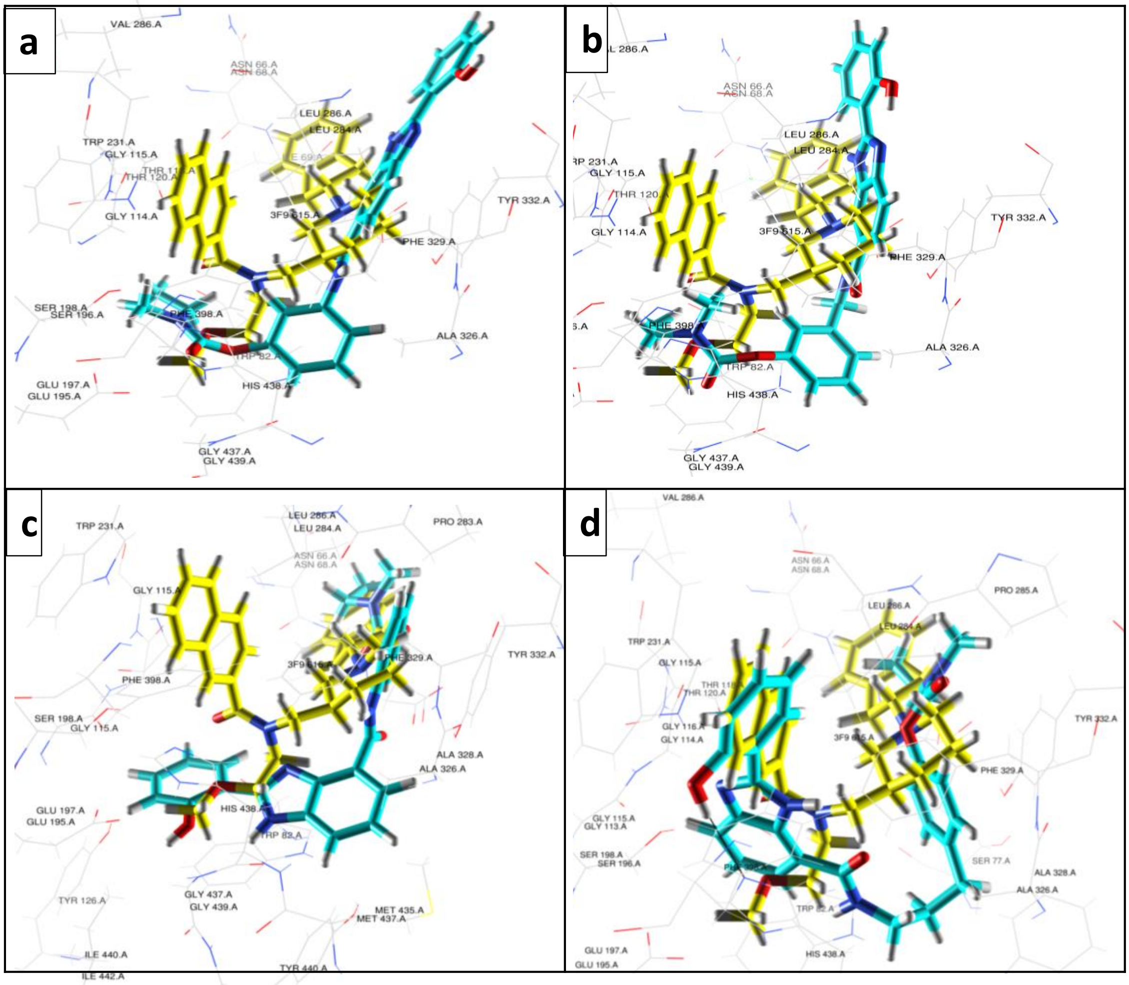

Figure S2. Docking results for RIV-BIM hybrids (blue) with hBuChE and pose comparisons with the original ligand (yellow, PDB code 4TPK): (a) **4a**; (b) **4b**; (c) **5a**; (d) **5d**.

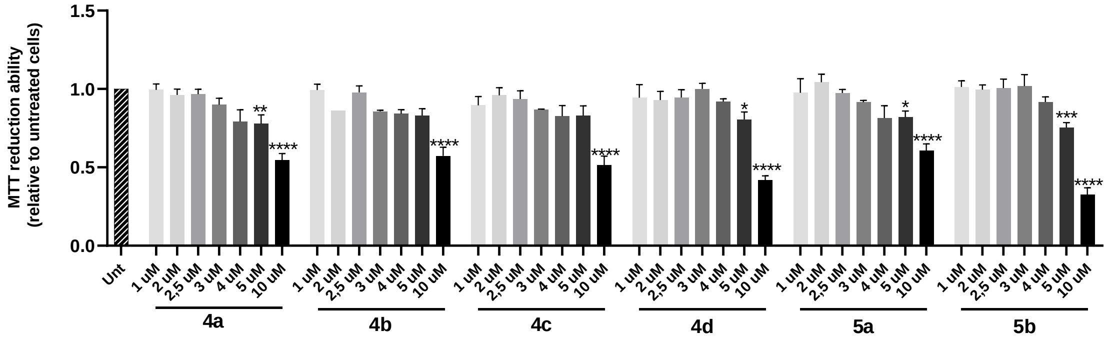

Figure S3. Dose-response screening of RIV-BIM compounds in SH-SY5Y cell line to select a non-toxic concentration. Cells were treated with different concentrations of the mentioned compounds for 24 h. To evaluate cell viability, MTT reduction assay was performed. Results are expressed relatively to SH-SY5Y untreated cells, with the mean ± SEM derived from eight different experiments. *$p < 0.05$; **$p < 0.01$; ***$p < 0.001$; ****$p < 0.0001$, significantly different when compared with SH-SY5Y untreated cells.
